# IDO-1 inhibitor INCB24360 elicits distant metastasis of basal extruded cancer cells in pancreatic ductal adenocarcinoma

**DOI:** 10.1038/s41401-022-01035-w

**Published:** 2022-12-14

**Authors:** Hada Buhe, Ji-xin Ma, Fang-zhou Ye, Chen-yun Song, Xin-yu Chen, Yang Liu, Huang Lin, Xu Han, Li-xiang Ma, Hexige Saiyin

**Affiliations:** 1 Department of Anatomy, Histology & Embryology, School of Basic Medical Science, Fudan University, Shanghai, 200032 China; 2 The School of Pharmacy, Fujian Medical University, Fuzhou, 350108 China; 3 State Key Laboratory of Genetic Engineering, School of Life Sciences, Fudan University, Shanghai, 200438 China; 4 Department of Pancreatic Surgery, Zhongshan Hospital, Fudan University, Shanghai, 200032 China

**Keywords:** pancreatic ductal adenocarcinoma, basal extrusion, IDO-1, INCB24360, liver metastasis, murine KPIC PDAC organoids

## Abstract

Neoplastic cells of non-immunogenic pancreatic ductal adenocarcinoma (PDAC) express indoleamine 2,3-dioxygenase 1 (IDO-1), an immunosuppressive enzyme. The metabolites of IDO-1 in cancers provide one-carbon units that annihilate effector T cells, and recruit immunosuppressive cells. In this study we investigated how IDO-1 affected the neoplastic cell behaviors in PDACs. Using multiple markers co-labeling method in 45-µm-thick tissue sections, we showed that IDO-1 expression was uniquely increased in the neoplastic cells extruded from ducts’ apical or basal domain, but decreased in lymph metastatic cells. IDO-1^+^ extruding neoplastic cells displayed increased vimentin expression and decreased cytokeratin expression in PDACs, characteristics of epithelial-mesenchymal transition (EMT). However, IDO-1 expression was uncorrelated with immunosuppressive infiltrates and clinicopathological characteristics of grim outcome. We replicated basal extrusion with EMT in murine KPIC PDAC organoids by long-term IFN-γ induction; application of IDO-1 inhibitor INCB24360 or 1-MT partially reversed basal extrusion coupled EMT. *Ido-1* deletion in KPIC cells deprived its tumorigenicity in immunocompetent mice, decreased cellular proliferation and macropinocytic ability, and increased immunogenicity. KPIC organoids with IFN-γ-induced basal extrusion did not accelerate distant metastasis, whereas inhibition IFN-γ-induced IDO-1 with INB24360 but not 1-MT in KPIC organoids elicited liver metastasis of subcutaneous KPIC organoid tumors, suggesting that lower IDO-1 activity accelerated distant metastasis, whereas IDO-1 was indispensable for tumorigenicity of PDAC cells and supports the survival of extruding cells.

## Introduction

Pancreatic ductal adenocarcinoma (PDAC) is nonimmunogenic and barely responds to immunotherapeutic agents, including PD-1/PD-L1 inhibitors, CTLA-4, and other immune checkpoint inhibitors [1–5]. Indoleamine 2,3-dioxygenase1 (IDO-1), a tryptophan metabolic enzyme with an immunosuppressive role, was upregulated in the neoplastic cells of PDACs and expressed in a context-dependent way [6–8]. However, the direct biological effects of IDO-1 on the neoplastic cell behaviors of PDACs are mainly unknown.

IDO-1 prevents the fetus from maternal T cell entrance and rejection [9–11]. IDO-1 expression in tumors, including neoplastic cells, macrophages, and dendritic cells, suppress T cell proliferation and natural killer cell activity, promotes regulatory T cell (Treg) and myeloid-derived suppressor cell (MDSC) development, and creates an immunosuppressive milieu [7, 12, 13]. IDO1 converts tryptophan to kynurenine, causing the depletion of tryptophan and increasing kynurenine in the tumor milieu [14]. Excessive kynurenine in the tumor milieu decreases the proliferation of cytotoxic T cells and accelerates the differentiation of Tregs [15]. In PDAC mice models immunized by the vaccine (GVAX), 1-MT treatments increased intratumoral cytotoxic T cell counts [7]. However, how IDO-1 directly affects tumorigenicity and metastasis is rarely reported.

Tryptophan, an essential amino acid, is critical for protein synthesis and 5-hydroxytryptamine and kynurenine precursors [16, 17]. Kynurenine pathway involved with reactive oxygen species (superoxide), one-carbon metabolism, synthesis of NAD(P)^+^ , synthesis of alanine, and carbons entrance (by a-ketoadipate) into the tricarboxylic acid (TCA) cycle; these are critical for tumor metabolism [16, 17]. A recent study showed that the increased IDO-1 in KPC cells accelerates one-carbon unit production from tryptophan utilized for a new purine nucleotide production [18]. Under low serine status, tryptophan is an alternative one-carbon source to support proliferation. The combined restriction of dietary serine, glycine, and tryptophan can sensitize PDAC cells to INCB24360 treatment, a kinase-selective IDO-1 inhibitor [18].

This work tested IDO-1 expression in human PDACs by multiple markers co-labeling methods in 45-µm-thick sections and IDO-1 roles in tumorigenicity and metastasis using murine KPIC cells and organoids. We showed that IDO-1 was uniquely expressed in apical or basal extruding cells in PDAC ducts but moderately in lymph and liver metastatic PDAC cells. Using IFN-γ induction, we replicated the basal or apical extrusion in KPIC PDAC organoids and found that inhibiting IFN-γ induced IDO-1 in KPIC PDAC organoids with INCB24360 decreased basal extrusion and EMT while promoting the metastasis of subcutaneous KPIC organoids tumor to the liver, indicating the unusual dual status of IDO-1 in tumorigenicity and metastasis of PDACs.

## Materials and methods

### Ethics statements

All mice purchased from Laboratory Animal Resource Center of Chinese Academy of Science were maintained in the standard animal rooms with air conditioning at the School of Basic Medical Science of Fudan University Animal Cores. School of Basic Medical Science of Fudan University and the Use Committee (Approval Series Number, 2020-0306-002) approved all animal procedures. All mice accessed enough food and sterilized water, and 4–5 mice were kept per cage. Mice were deeply anesthetized with isoflurane for surgery and sacrificed. The ethics committee approved the human ethics of Zhongshan Hospital of Fudan University (Y2017-012).

### High-resolution images and image processing

Nikon A1, Structured Illumination microscopy (SIM) (Nikon, Japan), and Zeiss 880 or 710 confocal microscopy (ZEISS, Jena, Germany) were applied to scan all Z-stacked and stitched images. ImageJ software (Fiji, NIH, Bethesda, MD, USA) was used to count cells and perform morphological analyses. Imaris 9.8 (Bitplane AG, Zürich, Switzerland) was used for the particle size and rendering displays.

### KPIC organoids and subcutaneous transplantation of organoids

After reaching 80% confluence in a 10 cm-dish, KPIC cells isolated from autochthonous KPIC tumor by our lab [19] were washed with 5 mL 0.01 M PBS and digested by addition of 5 mL 0.25% Trypsin–EDTA. After digesting for 3 min, KPIC cells were added with 5 mL DMEM containing 10% FBS to end digestion and suspended gently. The suspended cells were counted, and the KPIC cells were adjusted to 5000 cells/ organoid. Cells were centrifuged at 200 ×*g* for 2 min and the supernatants were discarded. The KPIC cells were re-suspended by a pre-dissolved Matrigel on ice. Matrigel and KPIC cell mixture (30 μL) was dropped on a well of 24 well plates. The droplets were observed under microscopy for gelation. After gelating the droplets, each well was supplied with 500 μL Pancreatic OGM Mouse Basal Medium (STEMCELL, USA) and moved to a cell incubator with 5% CO_2_. After fixation by 4% paraformaldehyde (PFA), the KPIC PDAC organoids were directly stained in the wells by the thick tissue staining protocol [20].

The KPIC organoids were transferred into a 10-cm dish with sterilized PBS by forceps from culturing wells. C57/B6 mice with 6–8 weeks were deeply anesthetized by isoflurane and then the skin under the upper left legs was cut with a scissor to expose the deep muscle layers, and the intact KPIC organoids Matrigel were transplanted into the hypodermis layers. After transplantation, the cut in the skin was closed by surgical sutures.

### Knockout *Ido-1* in KPIC cells

PX459 plasmid was utilized for clone guide oligos targeting the *Ido-1* gene. KPIC cells were plated and transfected with PX459 plasmids overnight. After transfection for 24 h, 1 μg/mL puromycin was supplied to screen cells for 3 d. Living cells were seeded in a 96-well plate by limited dilution to isolate monoclonal cell lines. The knockout cell clones are screened by Western blot and validated by Sanger sequencing.

### Immunofluorescence staining of cells, tissues

We selected 62 human PDACs from our previous tissue bank [21]. Immunostaining was performed as previously described [20]. Briefly, tissues were fixed in 4% PFA, put into 30% sucrose for dehydration, and sectioned into 45-μm-thick sections by cryostat for further immunostaining by antibodies mixture. Cells were fixed with 4% PFA, incubated with primary antibody overnight, and incubated with fluorescent secondary antibodies and DAPI for 3–4 h. TUNEL staining was done after immunofluorescent staining by following the manufactory’s protocol. Following antibodies were used for immunostaining or immunoblotting: IDO-1 (Cell Signaling Technology, D5J4E^TM^, rabbit, mAb #86630, 1:1000), CD45RA (BD Pharmingen, Clone: L48, 1:200), Foxp3 (Abcam, ab20034, 1:200), human VEGFR2/KDR/Flk-1 antibody (R&D, AF357, 1:200), E-Cadherin antibody (R&D, AF748, 1:100), CD11b antibody (Abcam, ab34216, 1:10000), CD8 antibody (Abcam, ab4505, 1:1000), CD11b antibody (Abcam, ab133357, 1:10000), purified mouse anti-human CD16 antibody (BD Pharmingen, Clone GO22, 1:200), anti-glucose transporter GLUT1 antibody (Abcam, ab115730, 1:1000), purified mouse anti-human CD3 antibody (BD Pharmingen, Clone SP34-2, 556648, 1:500), recombinant anti-Cytokeratin 19 antibody (Abcam, EP1580Y, 1:1000), 6-diamidino-2-phenylindole (DAPI, Sigma, D9542, 1:1000), IDO monoclonal antibody (Sigma, Clone mIDO-48, 1:500), IDO-1 monoclonal antibody (Enzo Life Sciences, Clone 2E2, 1:200), Vimentin antibody (Thermo Fisher Scientific, MA5-11883, 1:1000), Ki67 (EPR3610, Abcam, 1:1000), E-cadherin (Abcam, Ab1416, 1:500). Secondary antibodies: Alexa Fluro 488 donkey anti-mouse IgG (Invitrogen, A21202, 1:1000), Alexa Fluro 594 donkey anti-mouse IgG (Invitrogen, A21203, 1:1000), Alexa Fluro 594 donkey anti-rabbit IgG (Chemicon, A21207, 1:1000), Alexa Fluro 488 donkey anti-rabbit IgG (Invitrogen, A21206, 1:1000), Alexa Fluro 594 donkey anti-goat IgG (Invitrogen, A11058, 1:1000), Cy5 AffiniPure donkey anti-goat IgG (H + L) (Jackson, 705-175-147, 1:300), Cy5 AffiniPure donkey anti-mouse IgG (H + L) (Jackson, 715-175-151, 1:1000), Cy5 AffiniPure donkey anti-rabbit IgG (H + L), TUNEL (Thermo Fisher Scientific, C10618).

### Histology, immunohistochemistry, and cell counting

PFA (4%) fixed all tissues. A routine histological staining protocol did H&E staining. Immunohistochemistry was detected by following the provider protocol (Bosterbio, China). Briefly, the paraffin slices were boiled in sodium citrate buffer (pH=6.0) in a microwave for antigen retrieval after deparaffinized in xylene and gradient ethanol (100%–70%), treated with 3% H_2_O_2_ for annihilating endogenous peroxidase, incubated with primary antibodies at 4 °C overnight. The signals were detected with peroxidase streptavidin–biotinylated complex (SABC kit) and DAB/H_2_O_2_, and the slices were mounted after counterstaining with Hematoxylin. Cell counting was done on the cell counter panel of ImageJ software after imaging by Leica microscopy coupled with a DC500 camera.

### Drugs

IFN-γ (interferon-γ, mouse, Sigma, USA), 1-MT (26988-72-7, Sigma, USA), INCB24360 (S7910, Selleck, Shanghai, China), Dextran Tetramethylrhodamine (D1819, Sigma, MO, USA).

### Gemcitabine and abraxane treatments

Gemcitabine (Lilly, USA) was dissolved into sterilized PBS with a concentration of 100 mg/mL, and Abraxane (Celgene, USA) was suspended in Sodium Chloride Injection with a concentration of 100 mg/mL. Briefly, KPIC cells were cultured on a 6-well plate and allowed to incubate for 24 h before being treated with Gemcitabine and Abraxane. After another 36 h culture, cells were used for immunostaining after fixation with 4% PFA.

### Dextran uptake

After reaching 30%–40% confluence in culturing dishes, the cells were starved in sterilized PBS for 30 min, then supplied with fluorescent Dextran (10 ng/mL) and maintained in a cell culturing incubator for 30 min. After incubating, the cells were fixed and counterstained the nucleus with DAPI. After mounting, the cells were scanned by SIM microscopy with Z-stack. Imaris 9.7 were used for analyzing particles.

### Western blot analysis

The KPIC cells were digested in ice-cold RIPA buffer supplemented by phosphatase inhibitor PMSF for 30 min. A BCA assay kit counted the supernatant’s protein concentration (PD-BCA-125, BioThrive, Shanghai, China). Proteins (25 μg) were loaded in 12% gels for SDS-PAGE electrophoresis, and then the digested proteins were transferred from the gel to PVDF membranes (Millipore, MA, USA). The membranes were blocked with 5% skim milk in TBST (PBS and 0.1% Tween) and incubated with antibodies for 4 °C overnight. After incubation with primary antibodies, the membranes were rinsed with TBST 3 times for 5 min each time and then incubated with species-specific horseradish peroxidase-conjugated secondary antibodies (1:5000, Santa Cruz, Germany) for 60 min at room temperature. After 3 washes with TBST for 10 min each time, the membranes were developed with a super-sensitive enhanced chemiluminescence substrate kit (Biothrive Ltd, ECL-P-100, Shanghai, China) for visualization with a Tanon-4600 imaging system.

### Cell migration assays

The cell migration was measured by Transwell chambers (8-μm pore size, Corning #3422). KPIC cells pretreated with IFN-γ for 24 h and IFN-γ (for 24 h) + INCB24360 (for 12 h) (5.0 × 10^5^ cells/chamber) were plated in the upper chamber without FBS. The medium with 20% FBS in the lower chambers was placed and incubated for 24 h at 37 °C with 5% CO_2_. After incubation, all of the non-migrated cells on the upper surface were scratched with a cotton swab; the migrating cells on the lower surface were fixed with PFA (4%) and then stained with Hematoxylin for 1 h. The covering region of migrated cells was calibrated with ImageJ after stitching under microscopy. The cell covering region fraction values were used to estimate the cell migration. Nine randomly chosen fields were counted for each assay, or an average of nine fields was used to show the cell migration.

### Flow cytometry and cell morphometry analysis

KPIC cells treated with IFN-γ and INCB24360 were rinsed with 5 mL 0.01 M PBS and digested with 5 mL 0.25% Trypsin–EDTA. After digesting for 3 min, KPIC cells were supplied 5 mL 10% FBS DMEM to stop digestion, re-suspended gently, and the re-suspended cells were counted. And then, the cells were stained by PI for 5 min for flow cytometry analysis. After reaching 30%–40% confluence in dishes, KPIC cells were recorded by Phase Contrast microscopy for morphometry analysis.

### Statistical analyses

SPSS version 21.0 (SPSS Inc, Chicago, IL, USA) was used to analyze the survival significance of PDAC patients. GraphPad software was used to analyze in vivo and in vitro data. One-way ANOVA and *t*-test were used to test the differences, and the Kaplan-Meier method was used to estimate overall survival. The mean ± SEM/SD was used to present the data. *P* values < 0.05 were considered significant.

## Results

### IDO-1 was expressed highly in the invasive neoplastic cells and moderately in metastatic cells but not in the normal/precursor ducts in PDACs

**Fig. 1:**
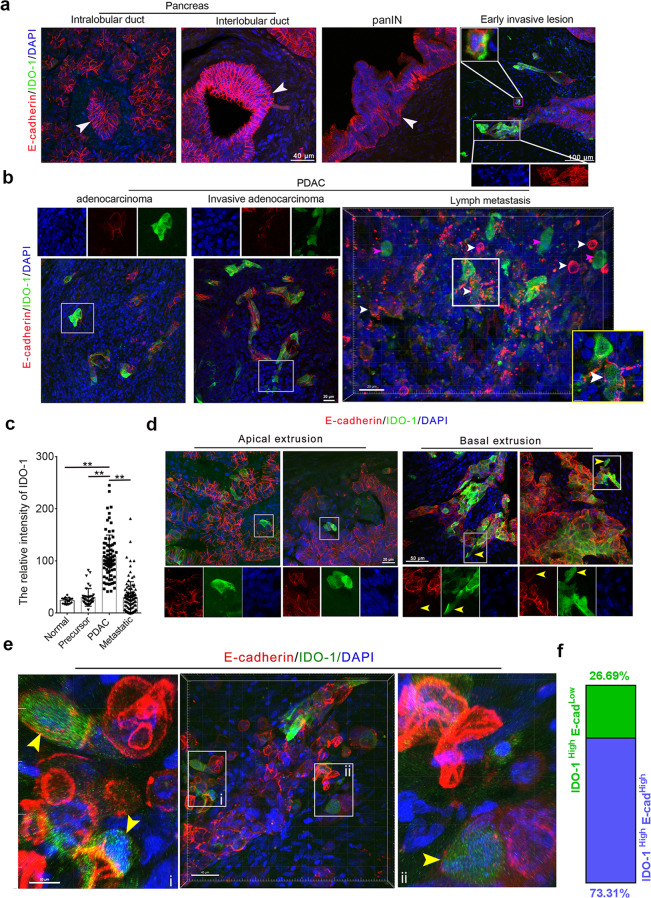
IDO-1 was highly expressed in the neoplastic cells with invasive characteristics and moderately in the metastatic cells but not the precursor lesion and normal ducts in PDAC tissues. **a** Immunostaining IDO-1 and E-cadherin antibodies in human PDACs. IDO-1 expression patterns in normal pancreatic ducts and precursor lesions (panINs) and early invasive lesions (white arrowheads, ducts; the lower panel in the left panel, E-cadherin staining, and nuclear morphology in the boxed region). **b** IDO-1 expression in the neoplastic duct, invasive lesions, and metastatic lymph lesions of PDACs (the upper panel, the split channels of the boxed region; the lower left in the right panel, the magnification of the boxed region). **c** Comparing the IDO-1 staining intensity in the neoplastic cells in PDAC or invasive lesions (sample size, *n* > 15) with lymph metastatic neoplastic cells and normal/precursors ducts (sample size, *n* = 4). Data, mean ± SD. One-way ANOVA. **P < 0.01. **d** Immunostaining IDO-1 and E-cadherin antibodies in human PDACs revealed IDO-1 is also exclusively upregulated in the cells with apical or basal extrusion (the lower panels, single channel of the boxed region). The 3D-rendered image revealed the morphology of basal extruding cells. **e**, **f** Significant fraction of IDO-1^+^ neoplastic cells in PDAC have discontinuous or uneven E-cadherin membrane staining. The circled area in the right and left panels (yellow arrowheads, IDO-1^high^, and E-cadherin^low^ neoplastic cells). The graph showed the counts of IDO-1^high^ and E-cadherin^low^ cells and IDO-1^low^ and E-cadherin^high^ cells in PDAC tissues.

To spatially show IDO-1 expressions in human PDACs, we co-immunostained 45-µm-thick PDAC tissues or noncancerous sections by E-cadherin, an epithelial marker, and IDO-1 antibodies in PDAC tissues. We observed that IDO-1 is not expressed in the intercalated duct, intralobular/interlobular ducts, pancreatic acini, and pancreatic islets and is barely detectable in pancreatic intraepithelial neoplasia (panINs) (Fig. 1a). We noticed that IDO-1^+^ neoplastic cells’ main population in PDAC is characterized by irregular basal domain and protruding signatures or detaching styles from the ducts (Fig. 1a). The neoplastic ducts of PDAC characterized by distorted/disrupted basal domain harbor multiple IDO-1^+^ neoplastic cells (Fig. 1b); a cell E-cadherin^+^ single with slender morphology showed a lower level of IDO-1 (Supplementary Fig. S1a); moderate or lower level of IDO-1 was detected in some E-cadherin^+^ metastatic cells in the lymphatic nodules and the liver, whereas barely detected in other E-cadherin^+^ cells in the fibrous capsule of lymphatic nodule (Fig. 1b, c, Supplementary Fig. S1b, S1c); E-cadherin^+^ cells in lymph nodules were also surrounded by several IDO-1^+^ and E-cadherin^-^ large cells with long processes (Fig. 1b and Supplementary Fig. S1a). Notably, the neoplastic cells extruded from the apical or basal domain uniformly expressed higher levels of IDO-1 (Fig. 1d). In the Z-stacked images of immunostaining that visualized an intact cell, we noticed that a significant fraction of IDO-1^+^ neoplastic cells, especially the neoplastic cells with extruding patterns, have a lower level of E-cadherin. E-cadherin staining signals are discontinuously distributed throughout the cellular membrane compared to IDO-1 weak or negative neoplastic cells in the duct, and IDO-1^+^ and E-cadherin^low^ cells account for 26.7% (67/251) (Fig. 1e, f). Our observation indicated that IDO-1 expression is preferentially upregulated in neoplastic cells with invasive and metastatic signatures and context-dependent in tissues.

The invasion and metastasis of cancer cells into the surrounding tissues and targeted organs paralleled inflammation, in which the tango of immunosuppressive cells and activated immune cells harness the balance of immune reaction [22]. Previously, we found that IFN-γ induced IDO-1 in murine macrophage-related M1-like macrophages with larger body size, enhanced phagocytic ability, and suppressed cellular motility and IL-1β secretion [23]. To characterize IDO-1^+^ and E-cadherin^-^ cells in invasive and metastatic lesions, we stained human PDACs with abundant immune cell infiltrates by E-cadherin, IDO-1, and CD45RA antibodies, an agranulocyte marker. IDO-1^+^ and E-cadherin^-^ cells in metastatic lymph nodules and invasive regions characterized by a larger body and long processes are positive for CD45RA staining (Supplementary Fig. S2a); IDO-1^+^, E-cadherin^-^, and CD45RA^+^ cells are not present in non-metastatic lymph nodules and the surrounding region of precursor lesions or noninvasive duct with the intact and smooth basal surface (Supplementary Fig. S2a, S2b); IDO-1^+^ and CD45RA^+^ cells are uniformly larger than IDO-1^-^ and CD45RA^+^ cells, resembling M1-like macrophages (Supplementary Fig. S2c). CD11B/CD3, IDO-1, and E-cadherin antibodies immunostaining further revealed that IDO-1^+^ immune infiltrates are macrophages (Supplementary Fig. S2d), and CD3^+^ cells expressed a lower level of IDO-1 compared to CD11B^+^ cells (Supplementary Fig. S2e, S2f). Our findings indicated that IDO-1 expression in PDAC neoplastic and immune cells is associated with invasive and metastatic characteristics.

Consistent with large-scale data analysis [24], our data revealed that higher levels of IDO-1 in both neoplastic cells and immune cells were neither related to clinicopathological parameters that indicate grim outcomes of PDAC patients, including lymph metastasis, neuronal invasion, major vessels invasion, and Ki67 scores, nor correlated with the relapse or survival of patients (Supplementary Table S1).

### IDO-1^+^ neoplastic cells in PDACs showed EMT

**Fig. 2:**
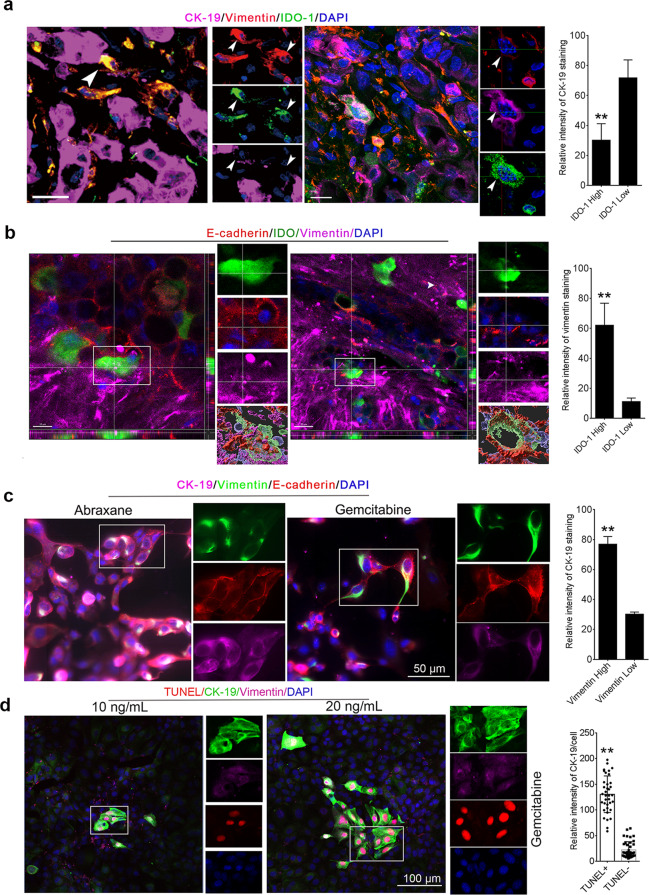
IDO-1^+^ neoplastic cells in the neoplastic ducts showed EMT characteristics. **a** CK-19, IDO-1, and vimentin antibodies immunostaining showed that IDO-1^+^ and vimentin^+^ cells in the neoplastic cells of PDAC that localized on the basal domain of ducts expressed a lower level of CK-19 (white arrows, basal extruding cells with EMT). Scale bar, 20 µm. Student *t* test. **P < 0.001. Data, mean ± SEM. **b** E-cadherin, IDO-1, and vimentin antibodies immunostaining showed that vimentin antibodies stained a few IDO-1^+^ and E-cadherin^+^ cells in PDAC tissues. Student *t* test. *P < 0.05, **P < 0.01. Data, mean ± SEM. **c** 1 ng/mL Abraxane and 5 ng/mL gemcitabine-treated KPIC cells for 12 h were stained by CK-19, E-cadherin, and vimentin antibodies. The results CK-19 immunostaining intensity in cells with higher vimentin levels and lower vimentin levels showed that KPIC cells with higher levels of vimentin also expressed higher levels of CK-19 and vice versa. Student’s *t* test. **P < 0.01. Data, mean ± SEM. **d** 5 ng/mL and 10 ng/mL gemcitabine-treated KPIC cells with over 80% confluence in dishes were performed CK-19, vimentin, and TUNEL staining. The measurement of staining intensity showed that TUNEL^+^ cells with intact cellular structure expressed higher levels of CK-19 compared to TUNEL^-^KPIC cells. Data, mean ± SD. *t* test, **P < 0.01.

Generally, apical epithelial extrusion is considered to separate and eliminate redundant or effector cells, while basal extrusion is the beginning of the invasion of surrounding tissues or metastasis [25, 26]. During basal extrusion, cancer cells undergo EMT, which is considered to drive distant metastasis [25]. Neoplastic cells of PDAC that undergo EMT expressed lower levels of E-cadherin [27]. To further find if IDO-1 expressing neoplastic cells with lower E-cadherin in PDAC also undergo EMT, we co-immunostained the PDAC tissues expressing IDO-1 with IDO-1, Cytokeratin-19 (CK-19), or E-cadherin, and vimentin antibodies and found that IDO-1^+^ neoplastic cells were located on basal domain or separated from duct expressing vimentin (Fig. 2a, b). Some IDO-1^+^ and vimentin^+^ neoplastic cells showed lower levels of CK-19 (Fig. 2b, left panel), which differs from gemcitabine or Abraxane induced EMT in PDAC cells that have higher levels of CK-19 (Fig. 2c), and higher levels of CK-19 expression in KPIC cells treated with gemcitabine were associated with an apoptotic phenotype (Fig. 2d). These data implied that IDO-1 expression is a specific signature of the extruding neoplastic cells in PDAC tissues that paralleled non-apoptotic EMT.

### Immune suppressive infiltrates did not preferentially approximate to IDO-1^+^ neoplastic cells in PDACs

IDO-1 is an immune suppressive enzyme that recruits Tregs and MDSCs and rejects cytotoxic CD8 cells in several solid tumors or placental tissues [28], whereas PDAC is nonimmunogenic [5]. To find the spatial relationship between IDO-1 high-expressing neoplastic cells with T cells, we stained the PDAC with CD3, IDO-1, and E-cadherin antibodies. After stitching a larger area with Z-stack by Confocal microscopy, we found that CD3^+^ cells did not preferentially surround IDO-1^+^ neoplastic cells, and some E-cadherin^-^ and IDO-1^+^, resembling macrophages, also co-existed with CD3^+^ cells (Supplementary Fig. S3a). We further detected the spatial relationship of IDO-1^+^ PDAC ducts with Tregs, immunostained with Foxp3 and CD45RA antibodies, and MDSCs, immunostained with CD11B and VEGFR2 antibodies in PDAC samples. The results revealed that the number of MDSCs in PDACs with high IDO-1 is higher than Tregs (Supplementary Fig. S3b). IDO-1, Foxp3, and E-cadherin antibodies’ staining data showed that Foxp3^+^ cells did not preferentially surround IDO-1^+^ neoplastic cells (Supplementary Fig. S3c). To find the global correlations between immune infiltrates and IDO-1 expression in pancreatic cancers, we analyzed 182 PDACs (TCGA) using CIBERSORT methods [29]. The results showed that IDO1 expression levels in pancreatic cancers were related to CD4^+^ memory T cells (*r*, 0.61), M1-like macrophages (*r*, 0.46), and CD8^+^ T cells (*r*, 0.19) but not other immunosuppressive cells, consistent with our immunostaining results (Supplementary Fig. S3d). Together, these findings implicate that the nonimmunogenic roles of IDO-1 in neoplastic cells might prevail over the immunosuppressive roles in PDACs.

### KPIC expressed IDO-1 and formed ductal organoids

**Fig. 3:**
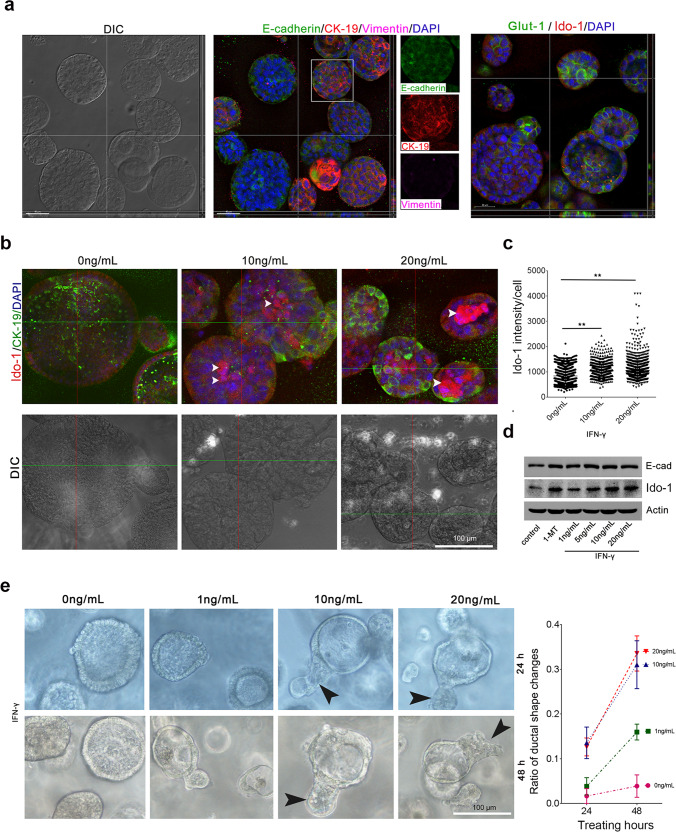
KPIC cells formed PDAC ductal organoids and expressed Ido-1. **a** The typical morphology of KPIC PDAC organoids taken by DIC microscopy and E-cadherin, CK-19, and vimentin antibodies co-immunostaining or Glut-1 and IDO-1 antibodies co-immunostaining at day 7. The spliced images in the middle panel from the boxed region showed that the PDAC KPIC organoids did not express vimentin. **b–d** Treating KPIC PDAC organoids with 0, 10, and 20 ng/mL IFN-γ for 24 h increases apical extrusion of IDO-1^+^ KPIC cells (**b**). IDO-1 expression levels in KPIC 2D culture after treatment with 0, 10, and 20 ng/mL IFN-γ and 20 µM 1-MT for 24 h (**c**, **d**). The intensity of the IDO-1 signal in each cell was measured by Imaris 9.7. One-way ANOVA, **P < 0.01. **e** Treating KPIC PDAC organoids with 0, 1, 10, and 20 ng/mL IFN-γ for 24 h and 48 h increased the distorting of ductal structure. Counting the ratio of ductal shape changes at 24 h and 48 h (replicates, *n* = 5; black arrowheads, the protruding basal cells).

Previously we reported genetically engineered mouse PDAC models (GEMMs), known as KPIC, harboring *LSL-Kras*^*G12D*^*; LSL-Trp*^*53R172H/+*^*; Ink*^*4flox/+*^*; Ptf1/p48-*^*Cre*^ [19]. We found that IDO-1 is expressed in the neoplastic cells that extrude into the duct lumen or metastasize into lymph in autochthonous KPIC (Supplementary Fig. S3e). We isolated tumorigenic KPIC PDAC cells from an autochthonous KPIC tumor, a typical epithelial tumor, undergoing EMT on gemcitabine and Abraxane treatments in 2D culture [19]. IDO-1 specifically increased in the extruded cells from the duct, and 2D culture models are unsuitable for studying IDO-1 roles in PDACs. Organoids, a 3D model in vitro, provides a tool to explore the IDO-1 role in PDAC cells [30]. Thus, we generated PDAC organoids by KPIC cells using Matrigel and Pancreatic OGM Mouse Basal Medium [31]. KPIC cells formed a typical ductal organoid that expressed cytokeratin 19 (CK-19) or Glut-1, E-cadherin but not vimentin (Fig. 3a). We have treated the KPIC ductal organoid with 0, 10, and 20 ng/mL IFN-γ for 24 h, a classical inducer of IDO-1. Contrary to a reported KPC cell line [18], the KPIC cell has a detectable level of IDO-1 without IFN-γ stimulation. A dose-dependent increase of IDO-1 expression was observed in IFN-γ treatment, and KPIC cells with a higher IDO-1 level mainly resided in the lumen of ductal organoids; these signatures are consistent with the observation in human PDAC and autochthonous KPIC tumors (Fig. 3b–d). A dose- and time-dependent ductal shape distortions were observed in KPIC organoids after 24 h and 48 h IFN-γ treatment (Fig. 3e). These findings indicated that KPIC organoids are suitable for studying the IDO-1 role in the extruding cells of PDACs.

### Deletion of *Ido-1* deprives the tumorigenicity of KPIC cells in immunocompetent mice but does not affect duct formation

**Fig. 4:**
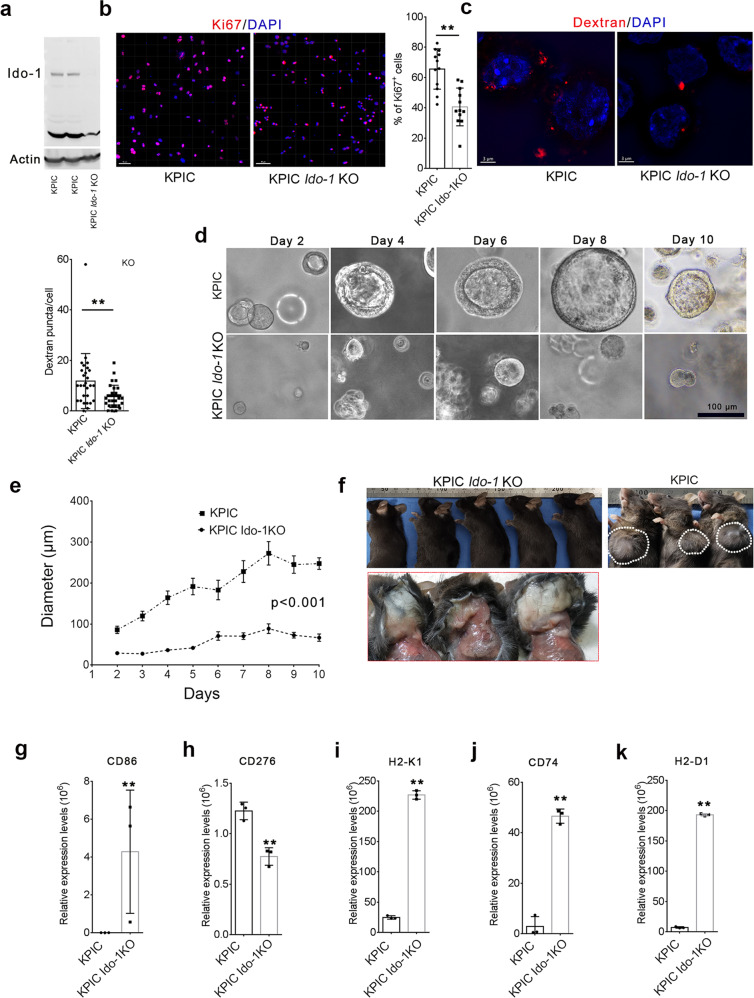
*Ido-1* knockout in KPIC reduced proliferation and macropinocytosis and deprived the tumorigenicity in the immunocompetent mice. **a** Testing IDO-1 expression in KPIC and KPIC *Ido-1* KO cells. **b** Representative images of Ki67 antibody immunostaining and count of Ki67^+^ cells in KPIC and KPIC *Ido-1* KO cells. Data, mean ± SD. *t* test, **P < 0.01. **c** Representative images of dextran uptake in KPIC and KPIC *Ido-1* KO cells after starvation and count of dextran vesicles in SIM Z-stacked images. Data, mean ± SD. *t* test, **P < 0.01. **d**, **e** Comparing the growth patterns and speeds of KPIC *Ido-1* KO PDAC organoids with KPIC organoids. Data, CI 95%. Two-way ANOVA. **f** KPIC *Ido-1* KO organoids did not form a subcutaneous tumor in C57/B6 mice after transplantation for 4 weeks (*n* = 7; white circled region, subcutaneous tumor of KPIC organoids). LS-MS data of KPIC and KPIC *Ido-1* KO cells showed that CD86 (**g**), CD276 (**h**), H2-K1 (**i**), CD74 (**j**), and H2-D1 (**k**) significantly changed after *Ido-1* knockout. Data, mean ± SD. *t* test; **P < 0.01. Repeats, 3 times.

To find if IDO-1 affects the polarization or extrusion behaviors of KPIC cells, we have knockout (KO) *the Ido-1* gene by targeted CRISPR/Cas9 system (Fig. 4a). One-carbon units derived from tryptophan facilitate serine and purine synthesis in tumors and support the proliferation of KPC cells under the limitation of serine supply [18]. Ki67 antibody’s immunostaining demonstrated that the proliferative KPIC cells in 2D dishes are higher than KPIC *Ido-1* KO cells (Fig. 4b). KPIC cells contain *Kras* mutation that enhances macropinocytosis in cells [32]. The dextran uptake ability of KPIC *Ido-1* KO cells was significantly decreased compared to KPIC cells in starvation (Fig. 4c). We generated ductal organoids with KPIC *Ido-1* KO cells. We observed that KPIC *Ido-1* KO cells could generate a duct but expand slower than KPIC organoids and noticed few luminal and basal extruding cells in both organoids without stimulation (Fig. 4d, e). KPIC cells are tumorigenic in immunocompetent mice in vein injection and orthotopic transplantation [19]. To test the tumorigenicity of KPIC *Ido-1* KO cells, we subcutaneously transplanted KPIC *Ido-1* KO organoids into immunocompetent C57BL/6 mice (*n* = 7), and found that KPIC *Ido-1* KO organoids did not form a subcutaneous tumor in C57BL/6 mice within 25 d after transplantation (Fig. 4f; Supplementary Table S2). To find the potential molecules involved in the loss of tumorigenicity in KPIC cells, we analyzed the whole proteomics of KPIC and KPIC *Ido-1* KO cells by LC-MAS. We found that CD86, a costimulatory molecule of T cells [33], is expressed in KPIC *Ido-1* KO cells but not in KPIC cells; CD276, an immune co-inhibitory molecule [34], is downregulated in KPIC *Ido-1* KO cells (Fig. 4g, h). Major or minor histocompatibility antigens (MHC) decides the immune rejection of transplanted cells [35, 36]. We also noticed that three MHC family protein expressions, including CD74, H2-D1, and H2-K1, increased in KPIC *Ido-1* KO cells compared to KPIC cells (Fig. 4i–k). These findings support that the host immune system might eliminate transplanted KPIC *Ido-1* KO organoids with higher immunogenicity.

### Long-term IFN-γ stimulation replicates basal extrusion in KPIC organoids, and IDO-1 inhibitors partially reverse basal extrusion

**Fig. 5:**
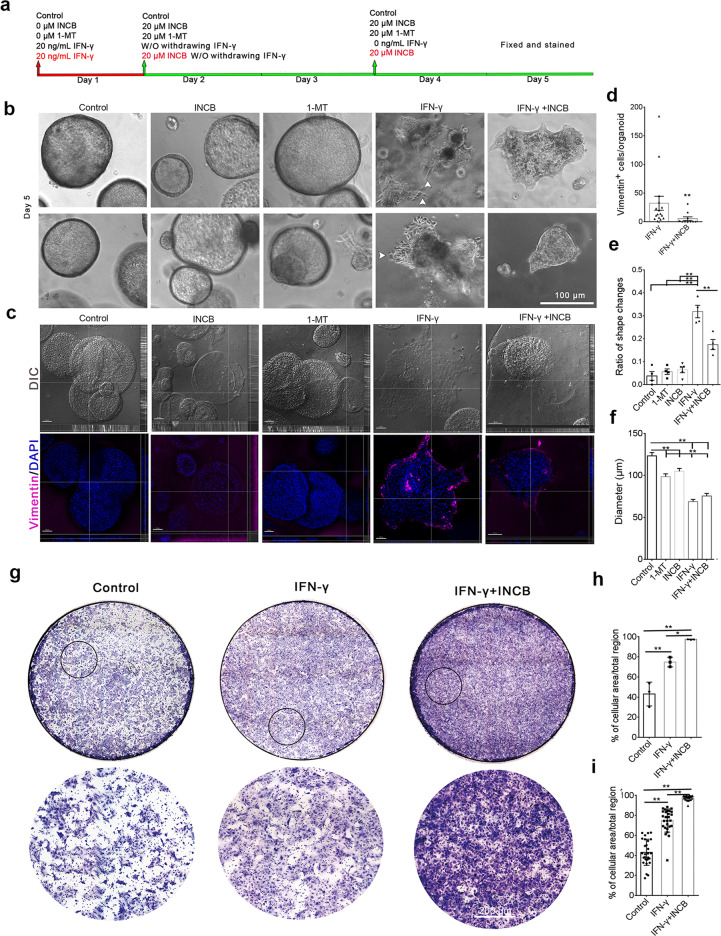
Long-term IFN-γ stimulation induces basal extrusion and EMT in KPIC organoids, and IDO-1 inhibitors partially reverse basal extrusion. **a** The schematic of IFN-γ, 1-MT, and INCB24360 treatments for KPIC PDAC organoids. **b** 20 ng/mL IFN-γ treatments for 4–5 d replicate basal extrusion in KPIC PDAC organoids (white arrows, the extruding cells with slender body). **c** Vimentin staining for KPIC PDAC organoids treated with 1-MT, INCB24360, IFN-γ, and IFN-γ + INCB24360 for 5 d. **d** The count of vimentin-positive cells in IFN-γ and IFN-γ + INCB24360 treated KPIC PDAC organoids. Student’s *t* test, **P < 0.01. **e** The ratio of KPIC PDAC organoid shape changes in five groups. One-way ANOVA, **P < 0.01. Repeats, 4 times. **f** KPIC PDAC organoid diameters in five groups. One-way ANOVA, **P < 0.01. Repeats, 4 times. **g** The images of Transwell assays in KPIC cells pretreated by IFN-γ, IFN-γ + INCB24360 groups showed that INCB24360 treatment enhanced the migration of IFN-γ treated KPIC cells. **h** Average cell coverage percentage in three groups. **i** Cell coverage percentage in 10× optical field; nine images for each well. Data, mean ± SD. *t* test; **P < 0.01. Repeats, 3 times.

Apical extrusion excludes aged, crowded, or dead cells in the epithelial duct system, whereas basal epithelial extrusion is the hallmark of cancer invasion and metastasis [25, 26, 37]. Based on a significant amount of IDO-1^+^ neoplastic cells that extrude from the basal domain of neoplastic ducts observed in human PDAC tissues, we extended the IFN-γ stimulating time to find if a long-term IFN-γ induction replicates basal extrusion in KPIC organoids. After treatment with one dose of 20 ng/mL IFN-γ for 4 d, plenty of cells have extruded from the basal surface of KPIC organoids, highly resembling IDO-1^+^ neoplastic cells protruding from human PDAC samples. KPIC cells protruding from the basal surface are slender (Fig. 5a, b), resembling EMT cells [38, 39]. IDO-1 inhibitors, 1-MT (a synthetic analog of tryptophan) and INCB24360 (a highly selective IDO-1 inhibitor) [9, 40], neither affect the ductal morphology nor induce basal extrusions (Fig. 5a, b). To find if the basal extruding cells have an EMT character, we stained the organoids induced by IFN-γ with a vimentin antibody and observed that basal extruding cells expressed vimentin (Fig. 5c). To find further if IFN-γ induced IDO-1 is related to basal extrusion of KPIC ductal organoids, we inhibited IDO-1 in KPIC PDAC organoids pretreated by 20 ng/mL IFN-γ for 24 h with INCB24360 for 4 d (Fig. 5a). INCB24360 partially inhibited basal protrusion, ductal distortion, and slightly reduced vimentin-positive cells (Fig. 5b, c). By counting the shape changes and organoid size, we found that 1-MT and INCB24360 did not affect organoids’ growth; IFN-γ significantly distorted the ductal structure, and INCB24360 significantly reduced the IFN-γ induced ductal distortion (Fig. 5d–f). Previously, we also observed that inhibiting IDO-1 in murine macrophages treated with IFN-γ increased migration. To find the role of IFN-γ induced IDO-1 in KPIC cell migration, we tested the migrating ability of KPIC cells by Transwell assay after pre-induction by 20 ng/mL IFN-γ for 12 h and inhibition with INCB24360 for another 12 h. The results showed that inhibiting IFN-γ-induced IDO-1 in KPIC cells by INCB24360 intensified the migration of KPIC cells (Fig. 5g–i). These findings implied that IFN-γ-induced IDO-1 is partially involved in basal extrusion and EMT, replicating the IDO-1^+^ extruding neoplastic cells in human PDAC, and indicating the dual roles of IDO-1 in basal extrusion and migration.

### Inhibiting IDO-1 in KPIC organoids with IFN-γ-induced basal extrusion elicits livers metastasis

**Fig. 6:**
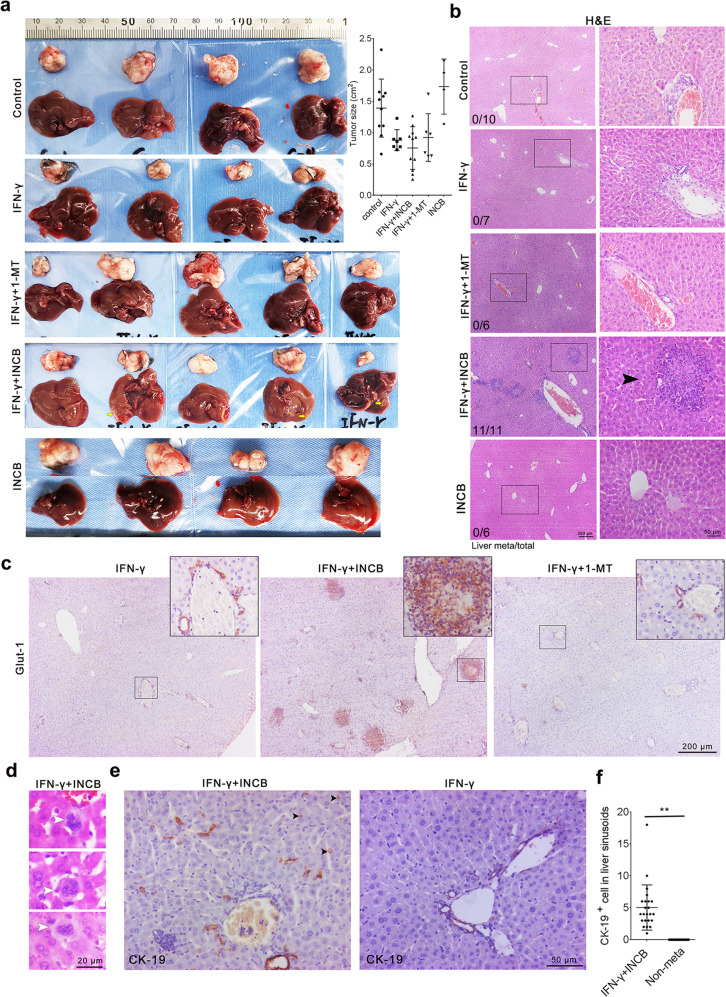
Inhibiting IDO-1 with INCB24360 in KPIC organoids with IFN-γ-induced basal extrusion elicits liver metastasis. **a** The gross anatomy of subcutaneous tumors and livers in mice subcutaneously transplanted KPIC PDAC organoids and pretreated with IFN-γ, IFN-γ + INCB24360, IFN-γ + 1-MT, INCB24360. The size of subcutaneous tumors in five groups. Data, mean ± SD. **b** H&E staining images of the livers from mice bearing KPIC organoid tumor pretreated with IFN-γ, IFN-γ + INCB24360, IFN-γ + 1-MT, INCB24360 (see also Supplementary Table S2; left corner: liver metastasis/total animals). **c** Glut-1 antibody staining images of the livers from KPIC PDAC organoids tumor pretreated with IFN-γ, IFN-γ + INCB24360, IFN-γ + 1-MT (see also Supplementary Table S2). **d** Multinuclear or large nuclear KPIC cells reside in the liver of the IFN-γ + INCB24360 group. **e, f** CK19^+^ cells in liver sinusoids of IFN-γ + INCB24360 group and count of CK-19^+^ cells in liver sinusoids (black arrowheads, neoplastic cells in sinusoid). Data, mean ± SD. Mouse, *n* = 3; total images: *n* = 23 (metastasis), *n* = 24 (non-metastasis).

Autochthonous KPIC tumors are invasive tumors that invade the liver and adrenal gland locally or metastasize to the spleen but not to the liver [19]. Basal extrusion and EMT are hallmarks of metastatic cancer cells [26, 38]. To find if IFN-γ induced-IDO-1 promotes the invasiveness or distant metastasis of KPIC tumors, we subcutaneously transplanted 5 groups of KPIC organoids pretreated with IFN-γ, INCB24360, IFN-γ + INCB24360, IFN-γ + 1-MT, and control into C57BL/6 mice after pretreatment for 5 days (Supplementary Fig. S4a, S4b; Supplementary Table S2). All five group organoids formed palpable subcutaneous tumors around 10 d, and the tumors in the control and INCB24360 groups are larger than others. At 25^th^ day, the largest tumor reached around 1.0 cm in size. Thus, after sacrificing the mice, we did a systemic pathological check for metastasis and invasion in tumor-bearing mice. Consistent with the enhancement of migrating ability by INCB24360, we noticed that several livers in IFN-γ + INCB24360 group have multiple noticeable yellowish lesions but not in other groups, resembling metastatic lesions (Fig. 6a). Pathologically, KPIC organoids in all five groups formed a subcutaneous tumor with rich stroma and ductal lesions, uniformly invading the dermis and muscular layer (Supplementary Fig. S4c–S4e). The subcutaneous tumors in all five groups are rare in CD8^+^ infiltrates, especially in the intratumor region (Supplementary Fig. S4f–S4h), and contain a decent amount of CD11b^+^ infiltrates in the surrounding stroma and the intratumor regions (Supplementary Fig. S4i–S4k). By histopathological analysis, we observed that the livers in IFN-γ + INCB24360 groups contain a large number of metastatic lesions with immune cells infiltration and Glut-1 expression (Fig. 6b, c; Supplementary Fig. S5a, S5b). The metastatic lesions in livers harbor a high percentage of CD11b^+^ cells but are rare in CD8^+^ infiltrates, and CD8^+^ cells mainly reside in the sinusoids but not in the metastatic lesions (Supplementary Fig. S6a, S6b). We also observed that some large KPIC cells with multiple or large nuclei reside in the liver (Fig. 6d), and a decent amount of CK^+^ cells are located in liver sinusoids of the IFN-γ + INCB24360 group livers (Fig. 6e, f). Comparing proteomics of the IFN-γ + INCB24360 group with other groups, we found that Claudin-3, a Zonula occludens, and inhibitor of carcinoma metastasis [41, 42], was dramatically reduced in IFN-γ + INCB24360 and INCB24360 treated KPIC PDAC organoids (Supplementary Fig. S6c). Of note, pnpla6 and Actr1b, the indicators of a favorable outcome in PDAC patients and other carcinomas (https://www.proteinatlas.org/), were also reduced in IFN-γ + INCB24360 and INCB24360 treated KPIC PDAC organoids.

Long-term IFN-γ treatment induced apoptosis in human PDAC cells with *Kras* and *p53* mutation [43]. The lower tumor mass in IFN-γ and IFN-γ + INCB24360 groups indicated that IFN-γ pretreatment might induce apoptosis in KPIC cells. To find the effect of IFN-γ induced IDO-1 on apoptosis and morphometry of KPIC cells, we treated cells with 20 ng/mL IFN-γ for 12 h and then with 20 µM INCB24360 for another 12 h or IFN-γ only. Non-treated cells were as control group. The apoptosis was detected by Flow Cytometry. The results showed that inhibiting IDO-1 in IFN-γ treated KPIC slightly increased the apoptosis of KPIC in 2D culture compared to IFN-γ treated KPIC or non-treated KPIC. IFN-γ treatment (20 ng/mL) also slightly increased the apoptosis of KPIC compared to non-treated KPIC (Supplementary Fig. S7a), consistent with other findings in PDAC cells [43]. The metastatic cancer cells often display a smaller minor axis and body size [44, 45]. The analysis of KPIC morphometry in control, IFN-γ, and IFN-γ + INCB24360 groups with optimized cellular density revealed that inhibiting IDO-1 in IFN-γ treated KPIC induced formation of smaller cells or cells with smaller minor axis compared to IFN-γ and control groups (Supplementary Fig. S7b–S7e). In 2D culture, these smaller and slender KPIC cells in IFN-γ + INCB24360 groups increased vimentin expression but not CK-19 expression compared to KPIC cells in IFN-γ and TUNEL^+^ apoptotic cells (Supplementary Fig. S8a–S8d). Collectively, our data indicate that inhibiting IDO-1 kinase activity in KPIC organoids with basal extrusion promotes the pro-metastatic features and seeding ability of KPIC subcutaneous tumors.

## Discussion

IDO-1 is a context-dependent enzyme with two important biological roles: immunosuppression and the provider of one carbon unit. IDO-1 in placental epithelial cells repels maternal cytotoxic T-cells and prevents the immune rejection of the fetus, and inhibiting IDO-1 in murine PDAC models strengthened vaccine-induced immunity via increasing T cell infiltration [7, 46, 47]. However, the failure of IDO-1 inhibitors in clinical trials did not support the dominant immunosuppressive effects of IDO-1 in solid tumors, especially in pancreatic cancers [48]. Using a modified thick-section staining method [20], we revealed that IDO-1 is uniquely increased in neoplastic cells that extrude from the basal or apical domain of PDAC neoplastic ducts, which undergo EMT. IDO-1 expression in PDACs did not relate to the immunosuppressive milieu. Depleting *Ido-1* in KPIC cells deprived its tumorigenicity in immunocompetent mice, and inhibiting IDO-1 by INCB24360 in IFN-γ induced organoids with basal extrusion elicits the liver metastasis of subcutaneous KPIC organoid tumors, supporting the dual roles of IDO-1 in tumorigenicity and metastasis.

Extra or aged cells in epithelial tissues, destined for apoptosis or die in normal adult tissues, are discarded via epithelial extrusion, the main way to maintain epithelial homeostasis [25, 49]. However, basal extrusion is a hallmark of beginning dissemination and metastasis in carcinoma, especially KRas mutated carcinoma cells [26, 50]. During extrusion, the carcinoma cells break the basal lining membrane, detach from the neighboring cells, enter a new milieu, and encounter strolling immune cells and a hostile environment for survival [38, 51]. The increases of IDO-1 expression in the apical or basal extrusion of human PDAC implied that the tumor milieu might drive IDO-1 expression in extruding cells. IFN-γ secreted by immune cells increased IDO-1 expression, induced EMT and apoptosis of PDACs, and inhibited their proliferation [43, 52, 53]. Our PDAC organoid data showed that IDO-1 is not the main driver of basal extrusion or EMT. EMT in pancreatic cancers also enhance chemoresistance but not metastasis [54–56]. IDO-1^+^ basal extruding neoplastic cells increased vimentin expression and decreased CK-19 expression, which differ from chemotherapeutic agents induced apoptotic EMT with higher CK-19. Cytokeratin increases cellular stiffness and inhibits invasive cancer cell behaviors, whereas vimentin drives metastasis or invasiveness [57, 58]. Metastatic cells are less stiff than non-metastatic cancer cells [59]. Thus, these IDO-1^+^ basal extruding cells showed a distinct signature of metastatic neoplastic cells in PDAC tissues.

Despite the controversy, EMT is considered to be indispensable for PDAC metastasis [55, 60–63]. We noticed that chemotherapeutic agent-induced apoptotic KPIC cells increased both cytokeratin and vimentin expression, differing from IDO-1 coupled non-apoptotic EMT cells with lower levels of cytokeratin. Higher levels of vimentin in breast, lung, and colorectal cancers are associated with severe metastasis and poor outcome [58, 64, 65]. The autochthonous KPIC and orthotopic KPIC tumors did not occur in liver metastasis within 30 d, whereas orbital injections cause large metastatic lung lesions in 20 d [19]. IDO-1 inhibition in IFN-γ-induced KPIC enhances the migrating ability and vimentin expression and creates more slender/smaller cells in 2D culture, supporting that IDO-1 inhibition accelerates IDO-1 expressing KPIC to disseminate. Lower levels of IDO-1 in the lymph metastatic PDAC cells and lower levels of CK-19 in basal extruding EMT cells with high IDO-1 support that the distant metastasis of human PDAC cells might depend on the changes of IDO-1 levels. Our findings also support that EMT with lower levels of CK-19 is necessary for basal extrusion and the survival of extruding cells in PDAC, whereas distant metastasis might need to decrease IDO-1 expression for strengthening motility and extravasation. Cell morphometry relates to the metastasis of PDAC cells and other tumor cells [66]. The smaller structure of extruding cells from neoplastic ducts did not provide the advantages of immediate metastasis, whereas smaller size coupled with lower IDO-1 acitivity might accelerate the metastatic behaviors of KPIC cells.

IFN-γ induced IDO-1 in murine macrophages formed M1-like macrophages with larger body size, more pseudofilapodia, enhanced phagocytic ability, and suppressed IL-1β secretion [23]. In virus infection, inhibiting IDO-1 with 1-MT intensified inflammatory reaction via secreting proinflammatory cytokine. M1-like macrophages in the invasive and metastatic region milieu indicated that the M1-like macrophages with larger bodies might play an immunosuppressive role. The strong correlation of IDO-1 with M1-like macrophage in CIBERSORT indicated that the increase of M1-macrophage is a common phenomenon in PDACs. In the future, the roles of IDO-1 expressing immune infiltrates in PDAC progression and metastasis needs to be explored.

Our work revealed the dual roles of IDO-1 in the tumorigenicity and metastasis of PDACs, and may partially explain the failure of IDO-1-based clinical trials. The deletion of *Ido-1* in KPIC deprived its tumorigenicity, whereas inhibition of IFN-γ induced IDO-1 in KPIC organoids with INCB24360 decreased basal extrusion and EMT while increasing pro-metastatic features of subcutaneous KPIC organoids.

## Supplementary information

Extended material-R3-APS-22272-figure-table-methods

## Data Availability

LC-MS data are available to anyone for a reasonable request.
