# Supplementary material for: IDO-1 inhibitor INCB24360 elicits distant metastasis of basal extruded cancer cells in pancreatic ductal adenocarcinoma
Source: Acta Pharmacol Sin. 2022 Dec 14;44(6):1277–89. doi: 10.1038/s41401-022-01035-w (PMC10203339; doi:10.1038/s41401-022-01035-w)
Supplement: Supplementary file 1 — Extended material-R3-APS-22272-figure-table-methods [file 41401_2022_1035_MOESM1_ESM.pdf]

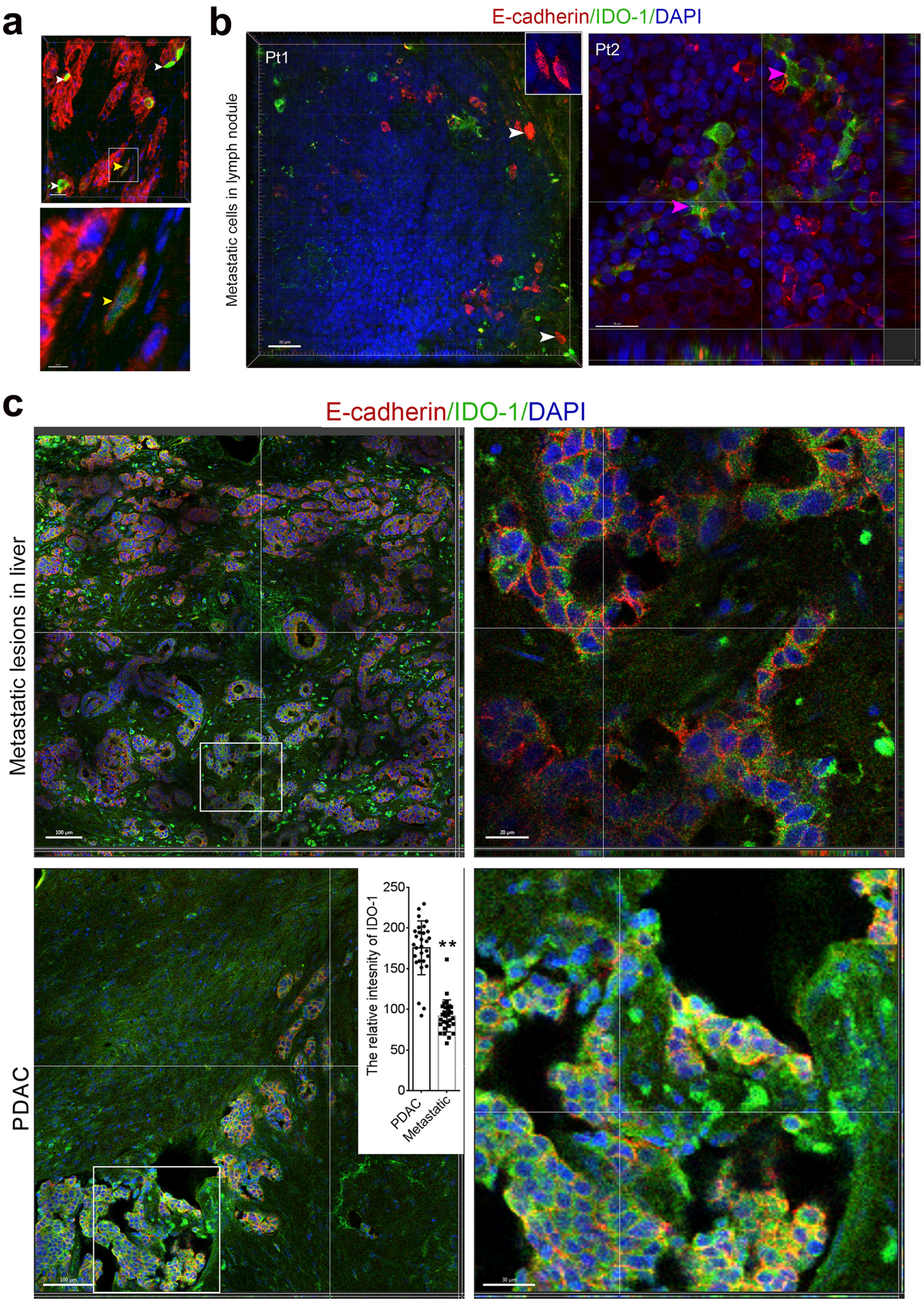
a
b
E-cadherin/IDO-1/DAPI
Pt1
Pt2
Metastatic cells in lymph nodule
30 μm
c
E-cadherin/IDO-1/DAPI
Metastatic lesions in liver
100 μm
20 μm
PDAC
100 μm
30 μm
The relative intensity of IDO-1
250
200
150
100
50
0
**
PDAC
Metastatic

Fig. S2-R2

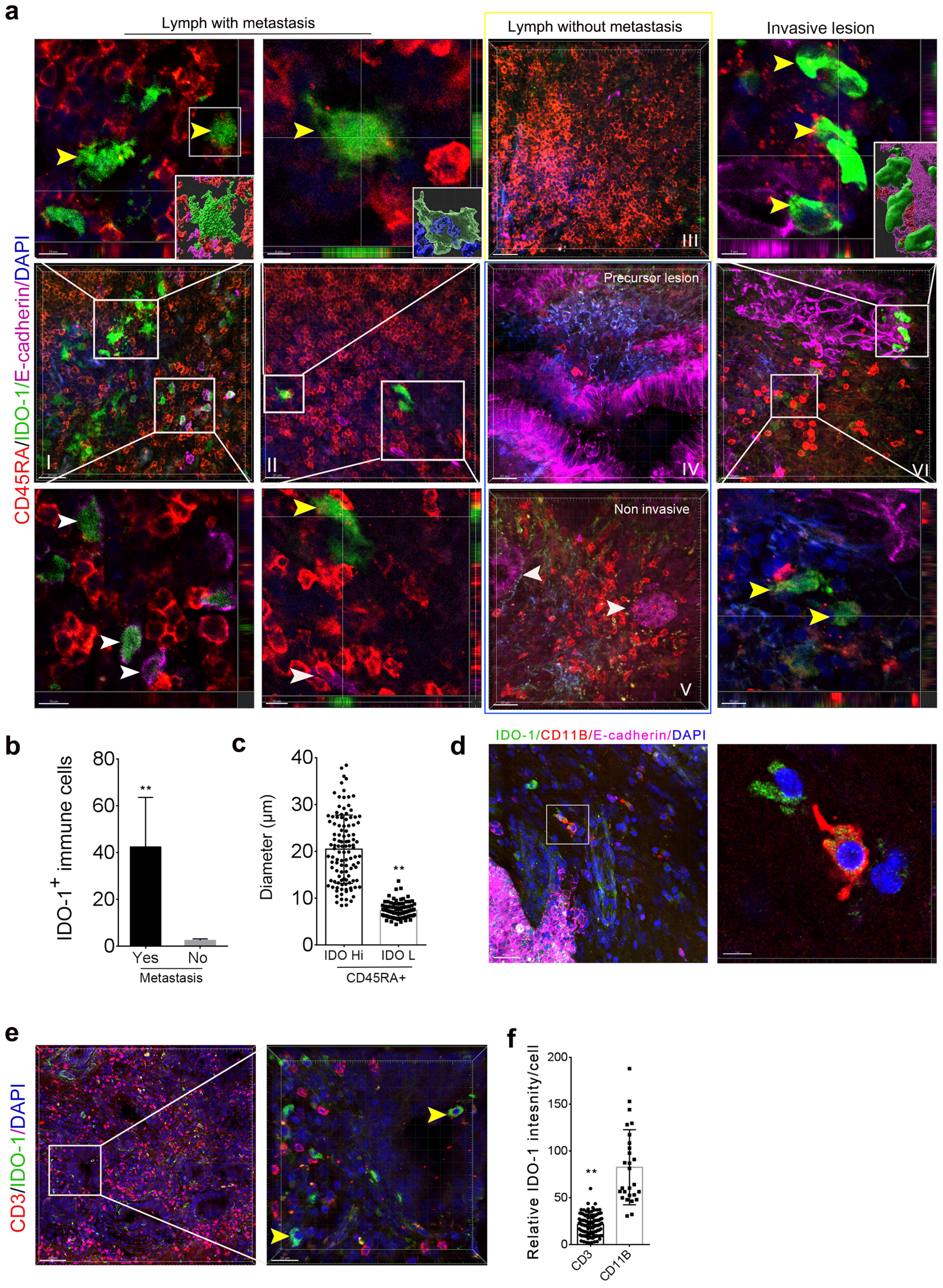
a
Lymph with metastasis
Lymph without metastasis
Invasive lesion
CD45RA/IDO-1/E-cadherin/DAPI
Precursor lesion
Non invasive
I
II
III
IV
V
VI
b
IDO-1+ immune cells
80
60
40
20
0
**
Yes
No
Metastasis
c
Diameter (μm)
40
30
20
10
0
**
IDO Hi
IDO L
CD45RA+
d
IDO-1/CD11B/E-cadherin/DAPI
e
CD3/IDO-1/DAPI
f
Relative IDO-1 intesnity/cell
200
150
100
50
0
**
CD3
CD11B

Fig. S3-R2

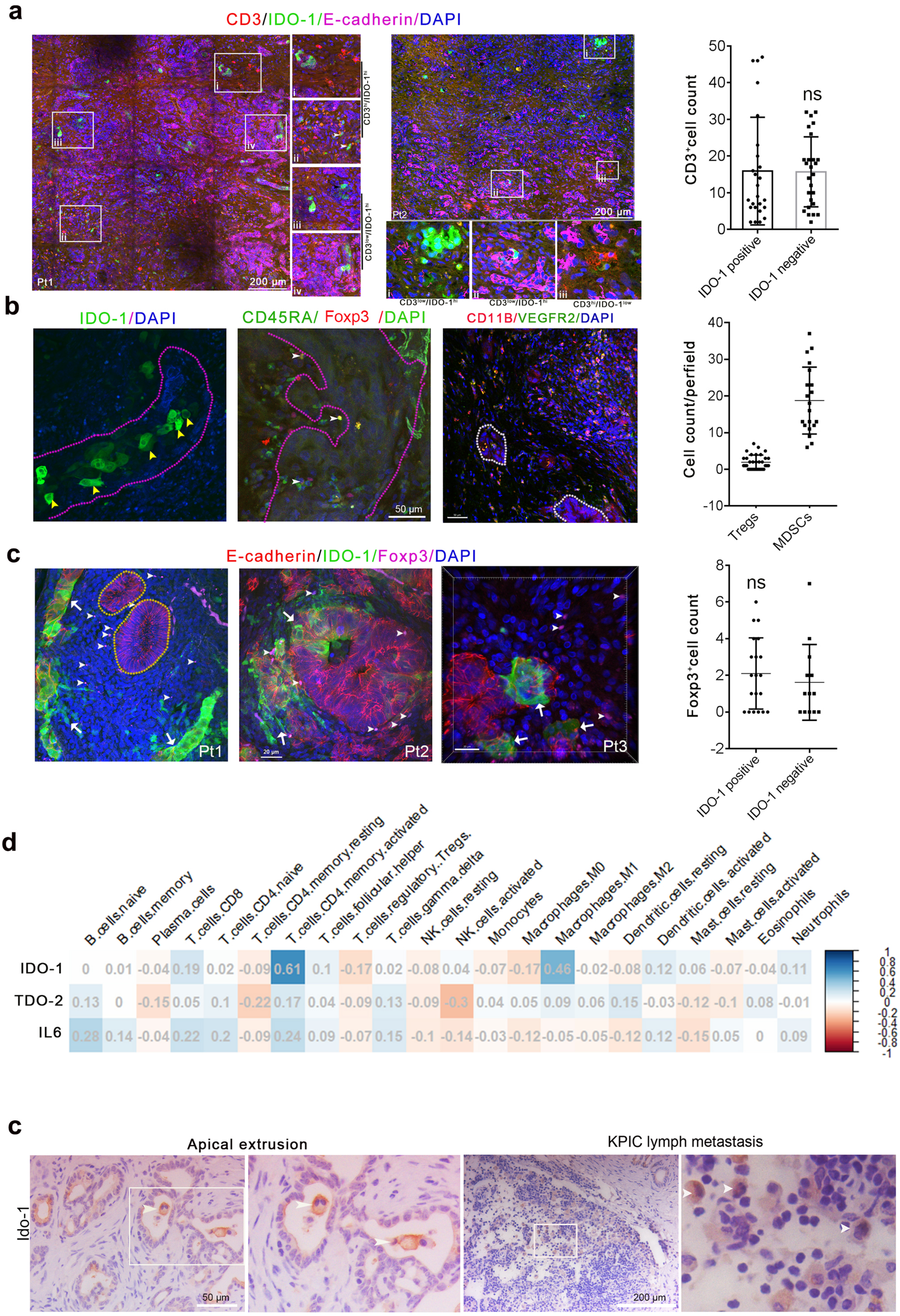
a
CD3/IDO-1/E-cadherin/DAPI
Pt1
200 μm
CD3hi/IDO-1hi
CD3low/IDO-1hi
Pt2
200 μm
CD3low/IDO-1hi
CD3low/IDO-1hi
CD3hi/IDO-1low
CD3+cell count
ns
IDO-1 positive
IDO-1 negative
b
IDO-1/DAPI
CD45RA/ Foxp3 /DAPI
50 μm
CD11B/VEGFR2/DAPI
Cell count/perfield
Tregs
MDSCs
c
E-cadherin/IDO-1/Foxp3/DAPI
Pt1
Pt2
Pt3
20 μm
Foxp3+cell count
ns
IDO-1 positive
IDO-1 negative
d
B.cells.naive
B.cells.memory
Plasma.cells
T.cells.CD8
T.cells.CD4.naive
T.cells.CD4.memory.resting
T.cells.CD4.memory.activated
T.cells.follicular.helper
T.cells.regulatory..Tregs.
T.cells.gamma.delta
NK.cells.resting
NK.cells.activated
Monocytes
Macrophages.M0
Macrophages.M1
Macrophages.M2
Dendritic.cells.resting
Dendritic.cells.activated
Mast.cells.resting
Mast.cells.activated
Eosinophils
Neutrophils
IDO-1 0 0.01 -0.04 0.19 0.02 -0.09 0.61 0.1 -0.17 0.02 -0.08 0.04 -0.07 -0.17 0.46 -0.02 -0.08 0.12 0.06 -0.07 -0.04 0.11
TDO-2 0.13 0 -0.15 0.05 0.1 -0.22 0.17 0.04 -0.09 0.13 -0.09 -0.3 0.04 0.05 0.09 0.06 0.15 -0.03 -0.12 -0.1 0.08 -0.01
IL6 0.28 0.14 -0.04 0.22 0.2 -0.09 0.24 0.09 -0.07 0.15 -0.1 -0.14 -0.03 -0.12 -0.05 -0.05 -0.12 0.12 -0.15 0.05 0 0.09
c
Apical extrusion
KPIC lymph metastasis
Ido-1
50 μm
200 μm

Fig. S4-R2

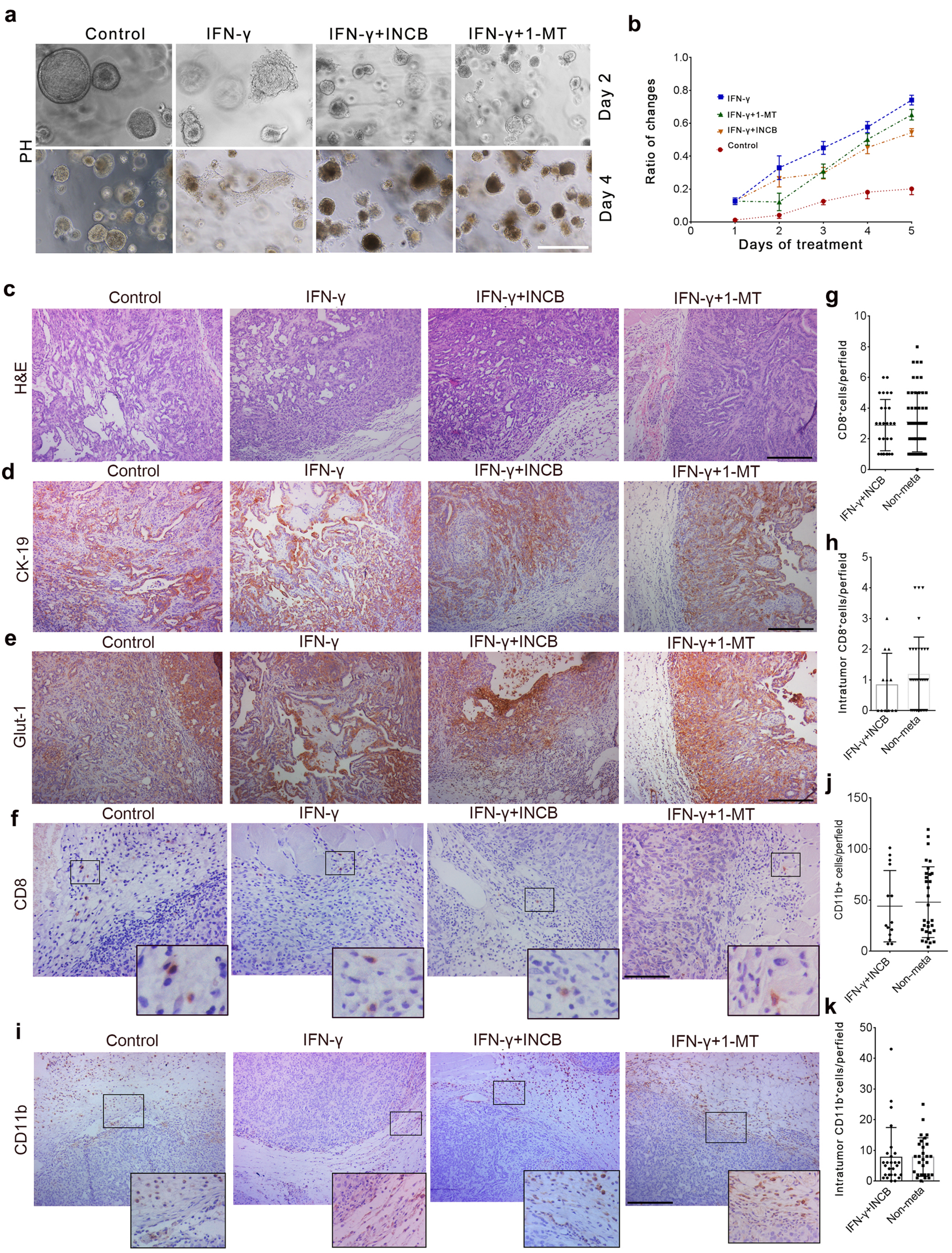
a
Control
IFN-γ
IFN-γ+INCB
IFN-γ+1-MT
PH
Day 2
Day 4
b
Ratio of changes
IFN-γ
IFN-γ+1-MT
IFN-γ+INCB
Control
Days of treatment
c
H&E
d
CK-19
e
Glut-1
f
CD8
g
CD8⁺cells/perfield
IFN-γ+INCB
Non-meta
h
Intratumor CD8⁺cells/perfield
j
CD11b+ cells/perfield
i
CD11b
k
Intratumor CD11b⁺cells/perfield

Fig. S5-R2

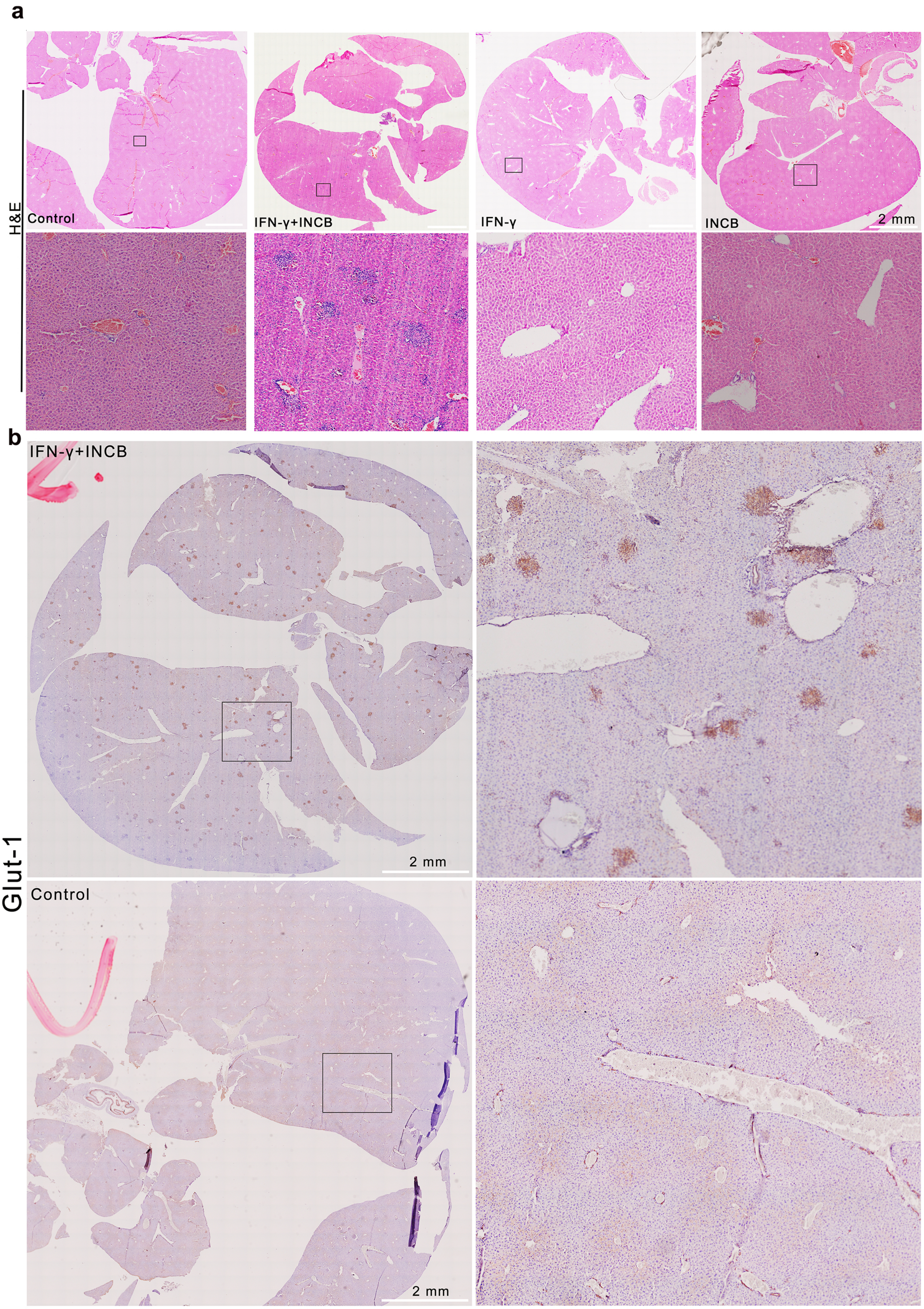

Fig.S4

Fig. S6-R2

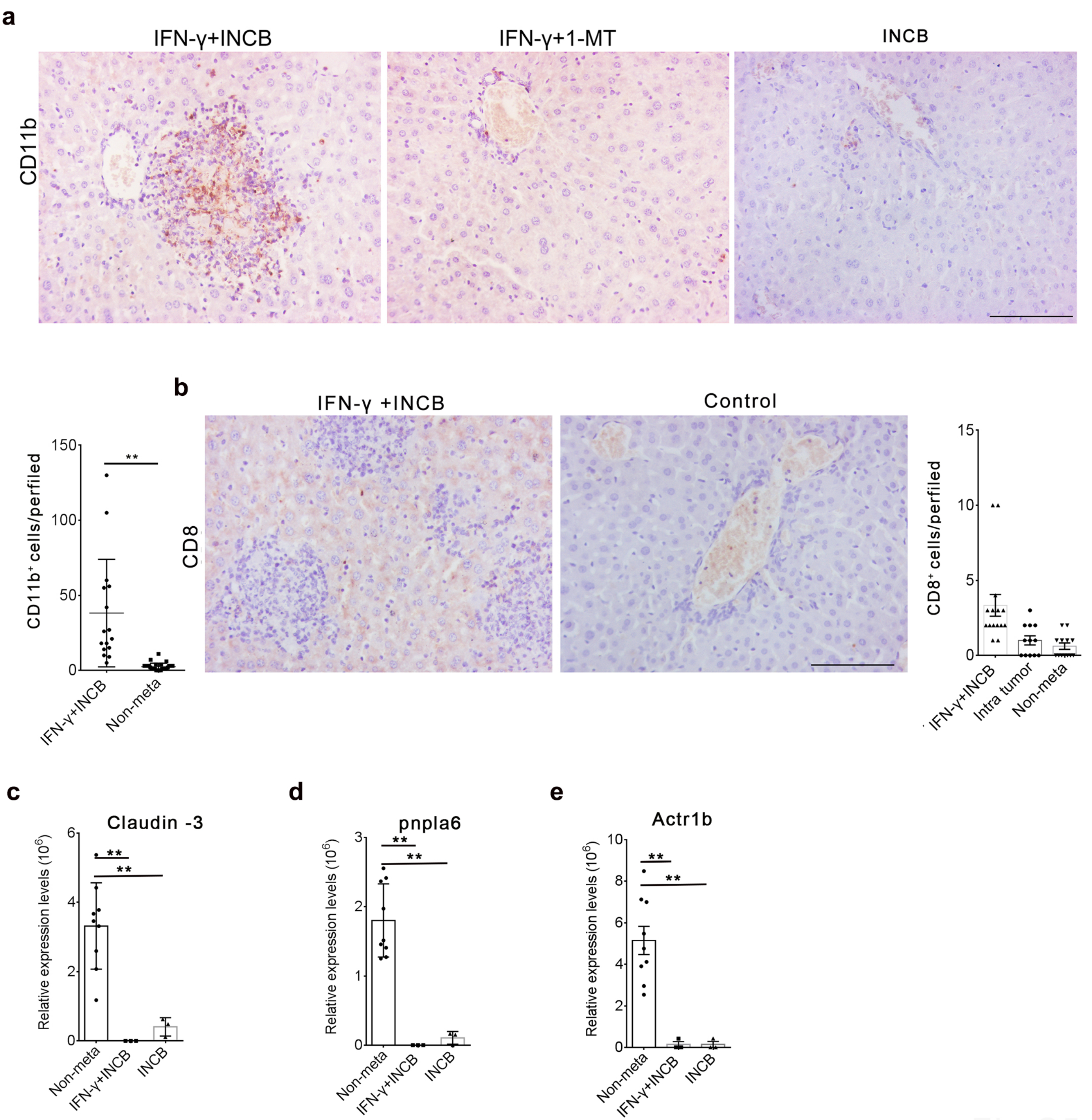
a
IFN-γ+INCB
IFN-γ+1-MT
INCB
CD11b
b
IFN-γ +INCB
Control
CD8
CD11b⁺ cells/perfiled
150
100
50
0
**
IFN-γ+INCB
Non-meta
CD8⁺ cells/perfiled
15
10
5
0
IFN-γ+INCB
Intra tumor
Non-meta
c
Claudin -3
Relative expression levels (10⁶)
6
4
2
0
**
**
Non-meta
IFN-γ+INCB
INCB
d
pnpla6
Relative expression levels (10⁶)
3
2
1
0
**
**
Non-meta
IFN-γ+INCB
INCB
e
Actr1b
Relative expression levels (10⁶)
10
8
6
4
2
0
**
**
Non-meta
IFN-γ+INCB
INCB

Fig. S7-R2

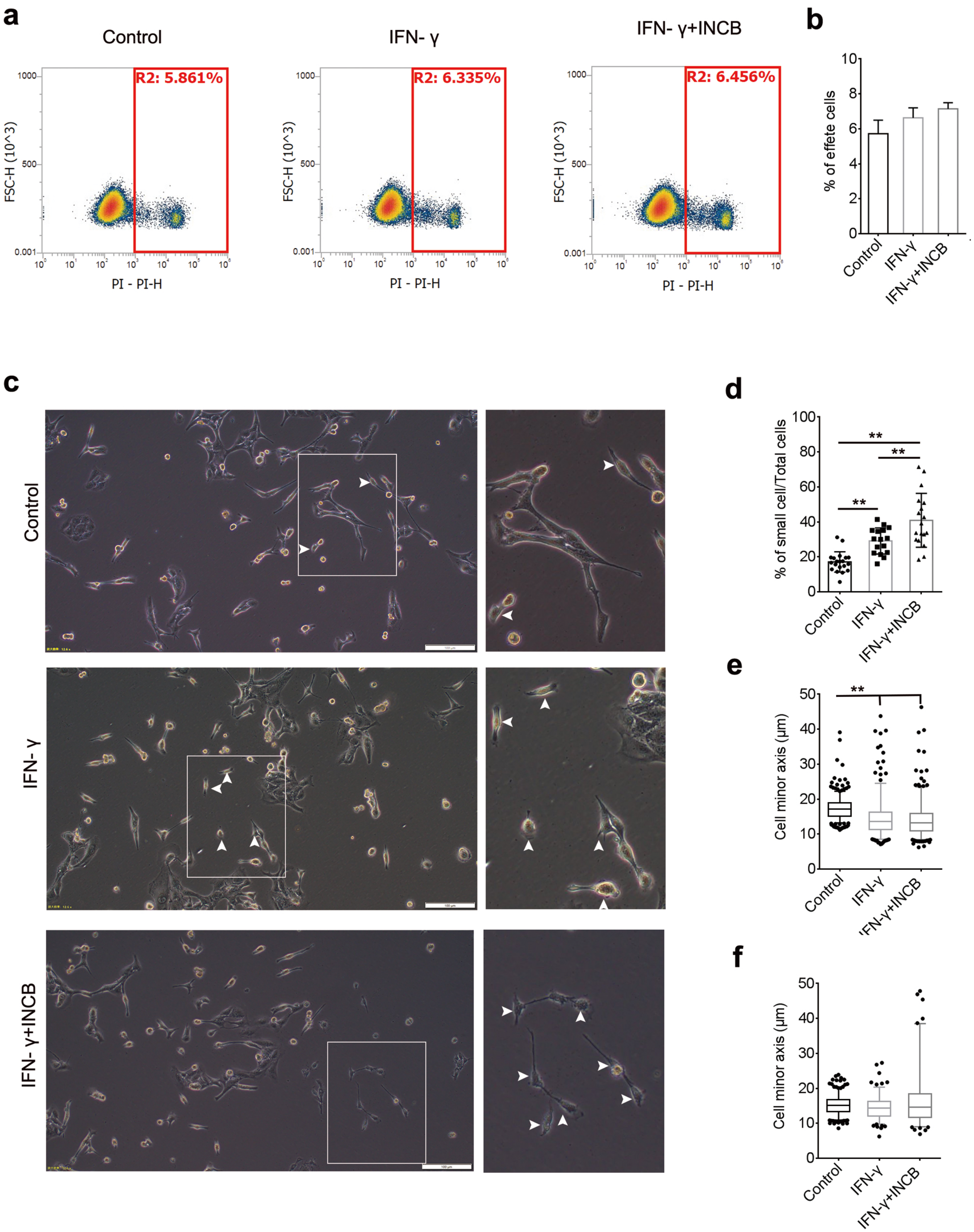
a
Control
IFN- γ
IFN- γ+INCB
R2: 5.861%
R2: 6.335%
R2: 6.456%
FSC-H (10^3)
PI - PI-H
b
% of effete cells
Control
IFN-γ
IFN-γ+INCB
c
Control
IFN- γ
IFN- γ+INCB
d
% of small cell/Total cells
**
e
Cell minor axis (μm)
f
Cell minor axis (μm)

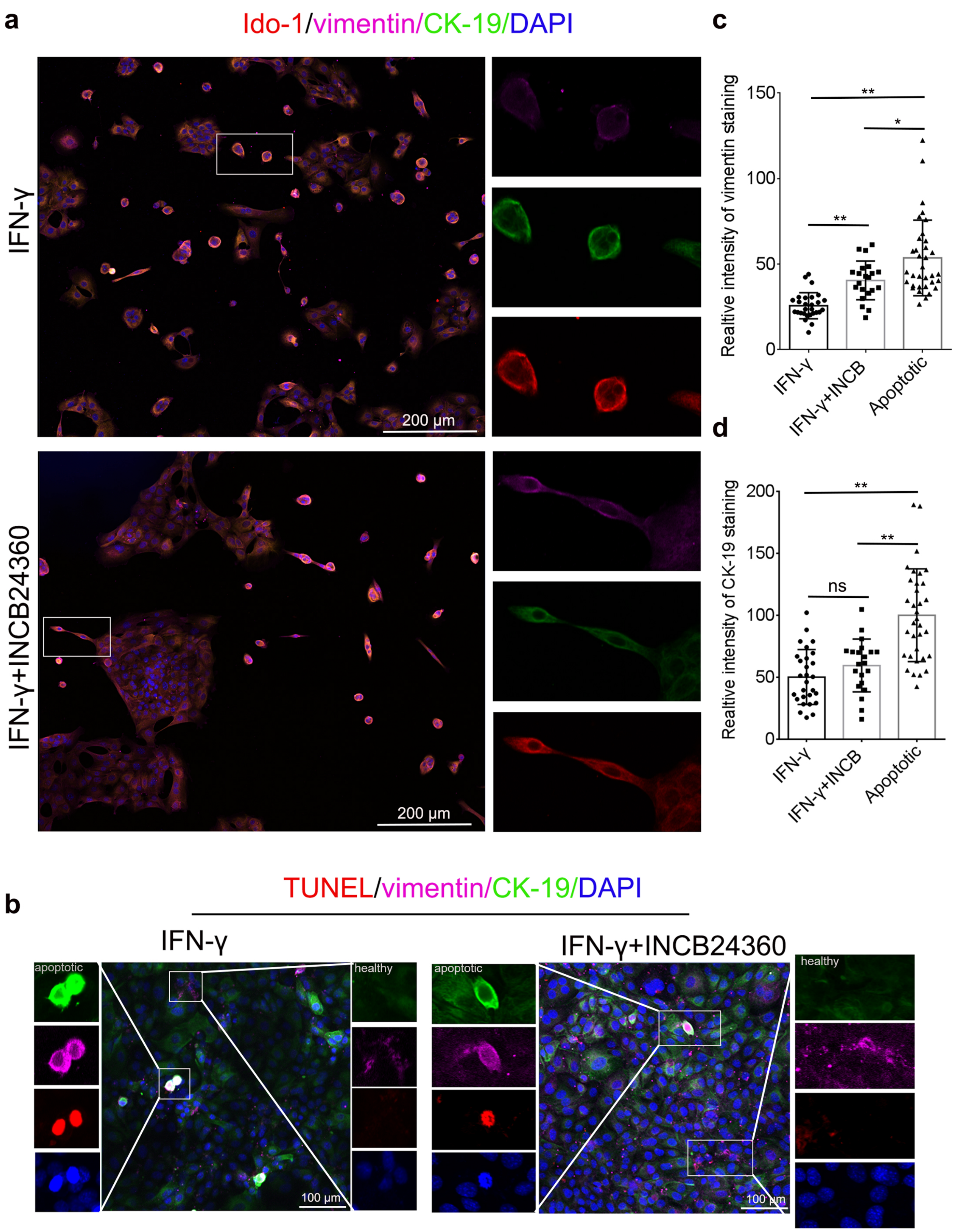
a
Ido-1/vimentin/CK-19/DAPI
IFN-γ
IFN-γ+INCB24360
200 μm
200 μm
c
Realtive intensity of vimentin staining
150
100
50
0
**
*
**
IFN-γ
IFN-γ+INCB
Apoptotic
d
Realtive intensity of CK-19 staining
200
150
100
50
0
**
**
ns
IFN-γ
IFN-γ+INCB
Apoptotic
b
TUNEL/vimentin/CK-19/DAPI
IFN-γ
IFN-γ+INCB24360
apoptotic
healthy
apoptotic
healthy
100 μm
100 μm

Extended data

Figure legends

Fig. S1. IDO-1 expression patterns in PDACs with lymph or liver metastasis

a. IDO-1 is moderately expressed in a single E-cadherin+ neoplastic cell with a slender body and nucleus (white arrowheads, high IDO-1 cells; pink arrowheads, a moderate IDO-1 cell).

b. IDO-1 and E-cadherin antibodies immunostaining in lymph nodules or the capsule of nodules in two patients (white arrows, the neoplastic cells on the capsule; pink arrows, IDO-1^+ and E-$cadherin^-$ cells with multiple processes).

c. IDO-1 and E-cadherin antibodies immunostaining in liver metastatic PDAC lesions revealed that metastatic neoplastic cells expressed lower level IDO-1 than its pancreatic lesions. Stuendet *t*-test, **, p<0.001.

Fig. S2. IDO-1 are highly and selectively expressed in M1-like macrophages in the invasive region or metastatic site of PDACs

a. The representative images of E-cadherin, IDO-1, and CD45RA staining in PDACs and the lymph nodules (I and II, a lymph nodule with metastatic cells; III, a lymph nodule without metastatic cells, IV, precursor lesion; V, noninvasive ducts; VI, invasive PDAC ducts with the disrupted basal surface; yellow arrows, immune cells; white arrows, metastatic cells in I and II, ducts in V). Sample size, n=10. The 3D rendered tomography showed the cellular shape (inner panels, the upper panels).

b. Comparing counts of IDO-1^{+} and CD45RA^{+} cells in lymph nodules with neoplastic cells with that in lymph nodules without metastatic neoplastic cells. 200 optical field. Data, mean ± SD. Student *t*-test. **, $p<0.001$.

c. Calibrating the size of IDO-1 high and IDO-1 low or negative and immune cells (the longest diameter of cells). Data, mean ± SD. *t*-test. **, $p<0.001$.

d. The representative images of CD11B, E-cadherin, and IDO-1 antibodies staining in PDAC tissues. The boxed region, the region of the right panel, and the right panel is a single layer view.

e. CD3 and IDO-1 antibodies staining in PDAC tissues showed that CD3+ have lower levels of IDO-1.

f. IDO-1 staining intensity in CD3+ and CD11B+cells of human PDACs. Data, mean ± SD. Student t-test. **, $p<0.01$.

Fig. S3. Immune suppressive infiltrates did not preferentially surround IDO-1^{+} PDAC ducts

a. Representative large area stitched images of two PDACs with Z-stack stained by CD3, E-cadherin, and IDO-1 antibodies staining showed the spatial relationship of CD3^{+} infiltrates with IDO-1^{+}or IDO-1^{-}PDAC ducts (the boxed region, the magnified regions). The count of CD3^{+} infiltrates surrounded an IDO-1^{+}or IDO-1^{-}PDAC duct. Student *t*-test. ns, non-significant.

b. The representative images of PDAC tissues with high levels of IDO-1 in neoplastic cells, abundant MDSCs, and rare Tregs (yellow arrowheads, apical extruding cells; white arrowheads, Tregs; pink [white in panel 3] circled region in panel 1 and 2, neoplastic ducts). MDSCs and Tregs count in PDAC tissues with high IDO-1 in neoplastic cells (PDAC samples, n=8; Total images: Tregs, n=31; MDSCs, n=20). Student *t*-test. **, $p< 0.01$

c. Foxp3, IDO-1, and E-cadherin antibodies immunostaining in three PDAC patients (Pt) showed that $Foxp3^+$ and E-cadherin$^-$ cells did not preferentially surround IDO-1$^+$ PDAC ducts (white arrowheads, $Foxp3^+$ cells; yellow circled, normal ducts; white arrows, the neoplastic duct). Comparing the $Foxp3^+$ cell counts that surrounded IDO1$^+$ PDAC ducts with that circled IDO-1$^-$ PDAC ducts (Patients, N>8; IDO-1$^+$ ducts, n=20; IDO-1$^-$, n=14). Student *t*-test; ns, non-significant

d. The correlations of *IDO-1* levels with immune cell populations in PDACs by using CIBERSORT methods.

e. IDO-1 antibody immunostaining images in autochthonous KPIC PDAC tissues and surrounding lymph nodules (white arrowheads, apical extruding cells in the left panel and lymph metastatic cells in the right panel; the boxed region, the magnified regions).

Fig. S4. Characterizing subcutaneous KPIC organoid tumors pretreated with IFN-γ, IFN-γ+INCB24360, IFN-γ+1-MT, INCB24360

a, b. The growth patterns and the shape-changing ratio of KPIC organoids before transplantation. Scale bar, 200 μm.

c. H&E staining of subcutaneous tumors showed the ductal growth patterns of KPIC organoid tumors and the invasion to muscular layers. Scale bar, 200 μm.

d, e. CK-19 and Glut-1 antibodies immunostaining subcutaneous KPIC tumors in IFN-γ, IFN-γ+INCB24360, IFN-γ+1-MT, INCB24360, and control groups. Scale bar, 200 μm.

f-h. $CD8^+$ antibodies staining of subcutaneous KPIC organoid tumors in IFN-γ, IFN-γ+INCB24360, IFN-γ+1-MT, INCB, and control groups; count of $CD8^+$ cells tumor regions (g, include stroma and infiltrated sites) and intratumor

(h,exclude stroma) in IFN-γ+INCB24360 group and other groups. Scale bar, 100 μm.

i-k. CD11b antibodies staining of subcutaneous KPIC tumors in IFN-γ, IFN-γ+INCB24360, IFN-γ+1-MT, INCB and control groups and count of $CD11b^+$ cells in tumor regions (j, include stroma and infiltrated sites) and intratumor (k, exclude stroma) of IFN-γ+INCB24360 group and other groups. The boxed region, the inner inserts. Sample size, N=5. Scale bar, 200 μm.

Fig. S5. The entire sectional views of the livers in IFN-γ, IFN-γ+INCB24360, IFN-γ+1-MT, and INCB24360 groups

a. The whole section views H&E staining in IFN-γ, IFN-γ+INCB24360, INCB24360, and control group.

b. A whole sectional views of Glut-1 immunostaining in IFN-γ+INCB24360, and control groups.

Fig. S6. Characterizing $CD8^+$ and $CD11b^+$ immune infiltrates in liver metastatic lesions

a. The representative images of $CD11b^+$ immune infiltrates in the liver of IFN-γ+INCB24360, IFN-γ+1-MT, INCB24360 groups; counting of CD11b+ cells. Data, mean± SD. Scale bar, 100 μm. Sample size, n=4.

b. The representative images of $CD8^+$ immune infiltrates in the liver of IFN-γ+INCB24360 and control groups and counting of $CD8^+$ immune infiltrates. Data, mean± s.e.m. Scale bar, 100 μm. Sample size, n=4.

c. Comparing Claudin-3 levels in IFN-γ+INCB24360 and INCB24360 treated KPIC organoids with other three groups, including control, IFN-γ and 1-MT. Data, mean± SD. *t*-test. **, $p<0.01$. 3 repeats.

d, e. Comparing pnpla6 and Actr1b levels in IFN-γ+INCB24360 and INCB24360 treated KPIC organoids with other three groups, including control, IFN-γ, and 1-MT. Data, mean± SD. *t*-test. **, $p<0.01$.

Fig. S7. Inhibiting IDO-1 in KPIC treated with IFN-γ increase cellular death and the number of smaller cell with metastatic signature

a. Detecting effete cells in the KPIC cells of IFN-γ, IFN-γ+INCB24360, control groups by Flow Cytometry. In IFN-γ group, KPIC cells have been exposed to 20 ng/mL IFN-γ for 24 h; In IFN-γ+INCB24360, the KPIC cells have been exposed to 20 ng/mL IFN-γ for 12 h and exposed to 20 μM INCB24360 for another 12h h without withdrawing IFN-γ. One-way ANOVA. Data, mean ± SD

b. Comparing the count of effete cells in IFN-γ+INCB24360 group with other groups. One-way ANOVA. Data, mean ± SD. **, $p<0.01$.

c. Representative living cells images of KPIC in IFN-γ, IFN-γ+INCB24360, control groups.

d. The count of smaller KPIC cells in IFN-γ, IFN-γ+INCB24360, control groups. One-way ANOVA. Data, mean ± SD. **, $p<0.01$.

e. The minor axis of separated single KPIC cells in IFN-γ, IFN-γ+INCB24360, control groups. One-way ANOVA. Data, mean ± SD. **, $p<0.01$.

f. The minor axis of packed KPIC cells in IFN-γ, IFN-γ+INCB24360, control groups. One-way ANOVA. Data, mean ± SD.

Fig. S8. Inhibiting IDO-1 in KPIC treated with IFN-γ increase vimentin expression but not CK-19

a. Vimentin, CK-19, and IDO-1 antibodies' immunostaining images of KPIC cells in IFN-γ, IFN-γ+INCB24360 group showed that the separated cells from

patched cells or slender cells expressed vimentin. The right panel is the magnified region of the boxed region with splicing channels.

b. TUNEL, vimentin, CK-19, and antibodies' immunostaining images of KPIC cells in IFN-γ, IFN-γ+INCB24360 groups showed that TUNEL+ KPIC cells dramatically increased CK-19.

c, d. Measuring vimentin (c) and CK-19 (d) levels in the separated single, slender and apoptotic cells of IFN-γ, IFN-γ+INCB24360 groups. One-way ANOVA. Data, mean ± SD. *, $P<0.05$. **, $p<0.01$

Supplementary methods

Isolation of KPIC cells from organoids

After scratching the KPIC organoids from the culturing well, the organoids were moved to a dish with DMEM/F12 on ice, blew with 1ml Pipette Tips three times, precipitated naturally for 1 minute， discarded supernatant， and then used the precipitant for LC-MS analysis.

Protein Digestion and Mass Spectrometry

Two milligrams of protein were mixed by buffer containing 8 m urea, 400 mm NH4HCO3 to 1.2 ml, and diluted with 5 mm dithiothreitol for 30 min at 50°C and alkylated by addition of 10 mm iodoacetamide. After 30 min of incubation in the light-proof room at RT, the first digestion was done by adding Lys-C at an enzyme/protein ratio of 1:100 and incubated for 4 h at 37°C. Subsequently, the digest was diluted by water to a final urea concentration of 1 m, and a second digestion with trypsin at an enzyme/protein ratio of 1:50 was done at 37°C overnight. Finally, the digestion was stopped with 5% formic acid. The digested protein was desalted by 200-ml Sep-Pak C18 cartridges (Waters Corp.), dried in vacuo, and stored at −20°C.

Peptide analysis was done on a reversed-phase nano-LC-coupled LTQ Orbitrap Classic, Discovery, or XL (Thermo Fisher Scientific). An Agilent 1200 series HPLC system was equipped with a 20-mm ReproSil-Pur 120 C18-AQ (Dr. Maisch, Ammerbuch-Entringen, Germany) trapping column (packed in-house, 100 μm inner diameter, 3-μm particle size) and a 400-mm ReproSil-Pur 120 C18-AQ (Dr. Maisch, Ammerbuch-Entringen, Germany) analytical column (packed in-house, 50 μm inner diameter, 3-μm particle size). As described previously (34), trapping was performed at 5 μl/min solvent C (0.1 m acetic acid in water) for 10 min, and elution was achieved with a gradient of 10–35% (v/v) of solvent D (0.1 m acetic acid in 80:20 acetonitrile/water) in 140 min with a total analysis time of 180 min. When performing the elution, the flow rate was passively split from 0.60 ml/min to 100 nl/min. Nanospray was achieved using a distally coated fused silica emitter

(360-μm outer diameter, 20-μm inner diameter, 10-μm tip inner diameter; constructed in-house) biased to 1.7 kV. Survey full-scan MS spectra were acquired from m/z 350 to m/z 1500 in the Orbitrap with a resolution of 60,000 at m/z 400 after accumulation to a target value of 500,000 in the linear ion trap with a target value of 30,000. The 10 most intense peaks were fragmented in the linear ion trap using collision-induced dissociation.

Data Analysis

All MS/MS spectra were centroided and merged to a single peak list file using MaxQuant (version 1.0.13.13) MaxQuant.org, which was searched using the Mascot search engine (version 2.2.0, Matrix Science, London, UK) containing a total of 67,344 entries (which includes contaminants and an equivalent number of decoy sequences) produced from the publicly available Arabidopsis database (The Arabidopsis Information Resource (TAIR9); June 2009; file name, TAIR9_pep_20090619) with carbamidomethylcysteine as a fixed modification. Oxidized methionine and phosphorylation (serine, threonine, and tyrosine) were searched as variable modifications. Searches were done with tryptic specificity allowing two miscleavages and an initial tolerance on mass measurement of 50 ppm in MS mode and 0.6 Da for MS/MS ions and a score cutoff of 20. The resulting .dat files were exported and filtered for a <1% false discovery rate at the peptide level using the in-house developed software Rockerbox (Version 1.1.0) (35). Relative quantification ratios of the identified phosphopeptides and protein were derived by MSQuant MSQuant.sourceforge.net (MSQ2.0b4, 2010-02-25) (36). Proteins were quantified with at least two unique nonphosphopeptides or a single peptide with a score no less than 60. The phosphopeptide ratio was normalized by dividing the ratio of its protein of origin. A p-value was generated using significance B, part of the MaxQuant package to determine if phosphorylation ratios for normalized peptides were significantly different. The changes were considered significant at a p value of 0.05 or less.

The raw data were analyzed by Proteome Discoverer (version 1.4, Thermo

Fisher Scientific) using an in-house Mascot server (version 2.3, Matrix Science, London, UK).(30)E. coli protein database (20161228, 4304 sequences) and Human protein database (20160213, 20 186 sequences) were downloaded from UniProt. The parameter for data search included up to two missed cleavage sites; 10 ppm mass tolerance for MS and 0.05 Da for MS/MS fragment ions; propionamidation on cysteine as fixed modifications; oxidation methionine as variable modifications. Additional enzyme-specific parameters were as follows: for citraconylated samples, Arg-C/P as the enzyme and protein N-terminal citraconylation, citraconylation on lysine as variable modifications; for Arg-C, Lys-C, or trypsin digested samples, Arg-C/P, Lys-C/P, or trypsin/P as the enzyme. For the analysis of wrong cleavage sites, the enzymes were changed to the corresponding semienzyme. The incorporated Target Decoy PSM Validator in Proteome Discoverer and the mascot expectation value was used to validate the search results and only the hits with FDR ≤ 0.01 and MASCOT expected value ≤0.05 were accepted for discussion. The mass spectrometry proteomics data have been deposited to the ProteomeXchange Consortium via the PRoteomics IDEntifications (PRIDE) partner repository with the data set identifier PXD007994.(31, 32)

LC-ESI-MS/MS analysis

LC-ESI-MS/MS analysis was performed using a nanoflow EASY-nLC 1000 system coupled to an LTQ-Orbitrap Elite mass spectrometer. A two-column setting was adopted for all analyses. Samples were firstly loaded onto an Acclaim PepMap100 C18 Nano Trap Column (5 μm, 100 Å, 100 μm id x 2 cm, (Thermo Fisher Scientific, Sunnyvale, CA, USA)) and then analyzed on an Acclaim PepMap RSLC C18 column (2 μm, 100 Å, 75 μm id x 25 cm. The mobile phases consisted of Solution A (0.1% formic acid) and Solution B (0.1% formic acid in ACN). The derivatized peptides were eluted using the following gradients: 2–40% B in 40 min, 40–90% B in 5 min, 90% B for 5 min, 90-0% B in 1 min, and 0% B for 9 min at a flow rate of 200 nL/min. Data-dependent analysis was employed in MS analysis: the 15 most abundant ions in each MS

scan were automatically selected and fragmented in HCD mode to achieve the high mass accuracy in MS/MS spectra. The resolution was set to 60 000 for MS and 15 000 for MS/MS, and the AGC target was set as 106 for MS and 5×104 for MS/MS. For MS/MS analysis, the isolation window was set as 2.0 Da, normalized collision energy as 35.0, activation time as 0.1 ms, and the starting mass as 100.0 Da. For each analysis, 0.5 μg of E. coli sample was injected.

Data analysis

Proteome Discoverer (Version 1.4, Thermo Fisher Scientific) using an in-house MASCOT server (Version 2.3, Matrix Science, London, UK) applied for analyzing raw data.

Supplementary table

S-Table1. The clinicopathological characteristics of pancreatic cancer patients and IDO-1 in immune cells and neoplastic cells

			IDO-1 in neoplastic cells			IDO-1 in immune cells		
Clinical variables		Count	positive	negative	Correlation	higher	lower	Correlation
Gender	Female	26（41.94%）	14	12.00	r=-0.286，p=0.024	9	17	r=-0.055,p=0.674
	Male	36（58.06%）	29	7		14	21	
Age	<60	18（29.03%）	5	13	r=-0.040，p=0.759	7	11	r=-0.016,p=0.904
	≥60	44（70.96%）	14	30		16	27	
Tumor stage	T1 +T2	39（62.90%）	27	12	r=0.004，p=0.978	14	24	r=0.023,p=0.861
	T3 +T4	23（37.09%）	16	7		9	14	
RLM	Yes	40（64.51%）	29	11	r=0.092，p=0.477	17	23	r=0.137,p=0.294
	No	21（33.87%）	14	8		6	15	
TNM	I+II	47（75.80%）	30	17	r=0.212，p=0.098	18	28	r=-0.052,p=0.693
	III+IV	15（24.19%）	13	17		5	10	
PNI	Yes	36（58.06%）	21	15	r=0.255，p=0.048	16	19	r=0.132,p=0.316
	No	26（41.93%）	22	4		7	19	
MVI	Yes	26（41.93%）	18	9	r=-0.051，p=0.693	12		r=0.124,p=0.341
	No	35（56.45%）	25	10		11	23	
Tumor vascular thrombus	Yes	22（35.48%）	13	9	r=-0.165，p=0.200	8	13	r=0.006,p=0.964
	No	40（64.51%）	30	10		15	25	
Resctional Marigin	R1	13（20.96%）	12	2	r=0.192，p=0.136	6	8	r=0.058,p=0.657
	R0	49（79.03%）	31	17		17	30	
CA19-9	Positive	55（88.70%）	37	18	r=0.127，p=0.327	22	32	r=-0.174,p=0.180
	Negative	7（11.29%）	6	1		1	6	
CEA	Positive	17（27.41%）	10	7	r=-0.140，p=0.276	5	12	r=-0.106,p=0.415
	Negative	45（72.58%）	33	12		18	26	
Ki67 index	<30%	29（46.77%）	22	7	r=-0.132，p=0.305	11	18	r=-0.004,p=0.973
	⩾30%	33（53.22%）	21	12		12	20	
PET-CT	<6.5	13（20.96%）	5	8	r=0.353，p=0.024	1	11	r=0.394，p=0.012
	>6.5	28（45.16%）	21	7		14	14	
	N.A	21（33.87%）						
Adjuvent therapy	Chemo	52（83.87%）	35	17	r=-0.055，p=0.671	20	31	r=0.148，p=0.256
	Chemo+radio	4（6.45%）	3	1		2	2	
	No	6（9.67%）	5	1		5	1	
Relapse	Yes	53（85.48%）	37	16	HR=1.243, 95%CI (0.86-2.27); p=0.48	21	32	HR=1.432, 95%CI (0.81-2.52); p=0.22
	No	9（14.51%）	6	3		2	6	
Survival	Live	33（53.22%）	20	13	HR=1.734, 95%CI (0.70-4.27); p=0.21	11	21	HR=1.277, 95%CI (0.60-2.70); p=0.53
	Died	29（46.77%）	23	6		12	17	

RLM, regional lymph metastasis; PNI, perineuronal invasion; MVI, major vascular invasion

Table S2. Summary of KPIC organoid transplanted mice

Groups	n	Syndromes	Death before scarifice	Liver metastasis
Control	11	Invasion to skin and muscles	1 (day 15)	0/10
IFN-γ	7	Invasion to skin and muscles	0	0/7
IFN-γ+INCB24360	11	Invasion to skin and muscles	0	11/11
IFN-γ+1-MT	6	Invasion to skin and muscles	0	0/6
INCB	6	Ascites and invasion to skin and muscle	2 (day 21 and day 23)	0/6
Ido-1ko	7	no tumors	0	0/7
